# Tau Protein Disrupts Mitochondrial Homeostasis in a Yeast Model: Implications for Alzheimer’s Disease

**DOI:** 10.1007/s12035-025-05255-z

**Published:** 2025-08-08

**Authors:** Yaisa Castillo-Casaña, Laura Kawasaki, Clorinda Arias, Hilario Ruelas-Ramírez, Soledad Funes, Norma Silvia Sánchez, María Guadalupe Códiz-Huerta, Laura Ongay-Larios, Roberto Coria

**Affiliations:** 1 Departamento de Bioquímica y Biología Estructural, Instituto de Fisiología Celular, UNAM, 04510 Cd Mex, México; 2 Departamento de Medicina Genómica y Toxicología Ambiental, Instituto de Investigaciones Biomédicas, UNAM, 04510 Cd Mex, México; 3 Departamento de Genética Molecular, Instituto de Fisiología Celular, UNAM, 04510 Cd Mex, México; 4 Unidad de Biología Molecular, Instituto de Fisiología Celular, UNAM 04510 Cd Mex, México

**Keywords:** Mitophagy, Retrograde response, Oxygen consumption, Magic pathway, 0N3R-tau

## Abstract

The microtubule-associated protein tau plays a central role in neurodegenerative diseases, called tauopathies, but the mechanism involved remains incompletely understood. Here, we used *Saccharomyces cerevisiae* as a model system to investigate the consequences of expressing the shortest human tau isoform 0N3R. After transfected, we detected widespread cellular distribution of tau and phosphorylation at key pathological residues involved in Alzheimer’s disease (Ser199/202). We also found that a portion of tau localizes within the mitochondrial matrix. The mitochondrial uptake of tau required a chaperone machinery, including Hsp104 and the Ssa1/Ydj1 bichaperone complex. Functionally, tau expression caused marked mitochondrial fragmentation, reduced oxygen consumption, and a decrease in membrane potential during stationary phase, indicating impaired mitochondrial function. This dysfunction activated the yeast retrograde signaling pathway. Importantly, tau expression enhanced mitochondrial clearance through mitophagy, both under nitrogen starvation and during stationary phase, and this effect was dependent on the retrograde response. Together, these findings demonstrate that tau expression in yeast perturbs mitochondrial homeostasis, triggering both compensatory nuclear signaling and increased mitochondrial turnover, adding evidence on the potential mechanisms involved in tau neurotoxicity.

**Graphical Abstract:**

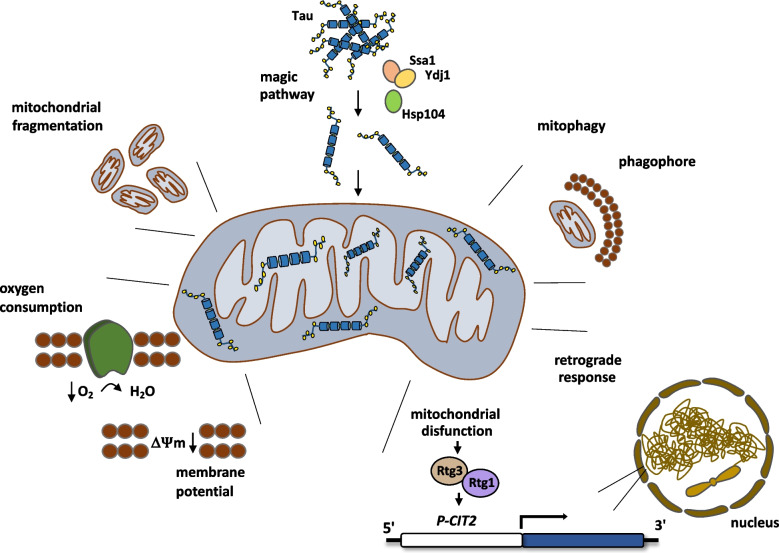

**Supplementary Information:**

The online version contains supplementary material available at 10.1007/s12035-025-05255-z.

## Introduction

Tau is a microtubule-associated protein primarily known for stabilizing neuronal microtubules, facilitating axonal transport. In patients with Alzheimer’s disease, tau becomes hyperphosphorylated and aggregates, forming neurofibrillary tangles in the neuronal soma. The aggregated form of tau loses its ability to bind to microtubules, its primary function. As a result, microtubule stability and axonal transport are disrupted, contributing to neuronal dysfunction [1]. Beyond its structural role, tau has emerged as a key contributor to mitochondrial dysfunction. Under pathological conditions, hyperphosphorylated tau accumulates and disrupts mitochondrial function through multiple mechanisms. Tau interacts directly with mitochondria, and its aggregates impair mitochondrial transport along microtubules, an essential process for delivering energy to neuronal synapses [1]. Disrupted transport leads to defective mitochondrial distribution and localized energy deficits, particularly in synaptic regions [2].

Phosphorylated tau also alters mitochondrial metabolism, reducing the activity of enzymes in the tricarboxylic acid cycle and impairing the electron transport chain, which compromises oxidative phosphorylation and decreases ATP production [3]. This metabolic dysfunction increases reactive oxygen species (ROS), exacerbating oxidative stress and mitochondrial damage [4]. Furthermore, accumulation of hyperphosphorylated tau affects mitochondrial morphology by promoting fragmentation and interfering with proteins that regulate mitochondrial fusion and fission, such as mitofusin (Mfn1/2) and dynamin-related protein 1 (DRP1) [5]. Fragmented mitochondria are less efficient and more prone to degradation, ultimately impairing synaptic function and contributing to cognitive decline [6, 7].

The human microtubule-associated protein tau has six isoforms resulting from alternative splicing of the MAPT gene, differing by the presence of N-terminal inserts (0N, 1 N, or 2 N) and either three (3R) or four (4R) microtubule-binding repeats [8]. While most studies have focused on the 2N4R tau isoform, our understanding on the contribution in the establishment of tauopathies of each of the six major tau isoforms expressed in humans remains limited. The shortest tau isoform (0N3R), characterized by the absence of N-terminal inserts and the presence of three microtubule-binding repeats (Fig. S1), is predominantly expressed during neurogenesis in fetal human brain [9]. In contrast, the adult human brain expresses all six tau isoforms, where the 0N3R accounts for about 10–20% of all tau species [10, 11].

The 0N3R protein plays a significant role in certain tauopathies, particularly those exhibiting an imbalance in tau isoform expression. Unlike the 4R isoforms, which include a fourth microtubule-binding domain conferring greater stability to microtubules, the 3R isoforms, like 0N3R, exhibit lower binding affinity, potentially leading to increased tau aggregation and pathology [12]. In disorders such as Pick’s disease, there is a predominance of 3R tau isoforms, which may promote the formation of pathological inclusions [13, 14].

In recent years, yeast has emerged as a suitable model for expressing human neurodegenerative disease–associated proteins and analyzing the potential mechanisms by which these altered proteins modify different cellular processes. Expression of amyloid-β (Aβ) peptides in yeast, for example, leads to aggregation and toxicity, enabling genetic screens that identify modifiers involved in endocytosis and cytoskeletal dynamics [15]. Similarly, expression of human tau in yeast recapitulates key pathological features observed in Alzheimer’s disease, including hyperphosphorylation, conformational changes, and aggregation [16]. In yeast, tau phosphorylation is regulated by the kinases Pho85 and Mds1, which are functional orthologs of the mammalian kinases GSK3β and CDK5. These kinases, among others, mediate tau hyperphosphorylation from the early stages of Alzheimer’s disease [14].

While there is evidence linking tau proteins to mitochondrial dysfunction, direct studies focusing on the mechanisms by which tau, and particularly the shorter 0N3R isoform, impacts on mitochondrial metabolism are currently lacking. In this study, we specifically investigate the effects of expressing the 0N3R isoform of the tau protein on mitochondrial homeostasis in yeast, making this the first report to directly address this relationship. Our findings reveal that several mitochondrial features, including morphology, dynamics, and bioenergetics, are significantly altered in the presence of 0N3R-tau, underscoring its potential to disrupt fundamental aspects of mitochondrial function.

## Materials and Methods

### Yeast strains, plasmids, and media

*Saccharomyces cerevisiae* strains used in this work are listed in Table S1. The primers used are shown in Table S2. The cDNA coding for human fetal tau isoform (0N3R) fused to the fluorescent protein GFP was obtained from the plasmid pCMV3-C-GFPSpark (Sino Biological, Cat: HG10058-ACG), and subsequently inserted into the episomal plasmid pVT100U (Addgene Cat No. 45054), which contains the constitutive *ADH1* promoter and the Ura3 marker. Untagged tau ORF was amplified by PCR and subcloned into pVT100U. The *CIT2* promoter (coordinates −898 to + 24) was subcloned into the episomal plasmid pRS415 (ATCC, Cat No. 87520) to yield the *P-CIT2* reporter. Plasmid pYX122 (Addgene Cat No. 45048) was used as recipient of mitochondrial version of mCherry (mCherry tagged with the F0 mitochondrial import sequence) replacing the GFP ORF. Yeast cells were grown in either YPD medium (1% yeast extract, 2% peptone, 2% glucose) or YPLac (1% yeast extract, 2% peptone, 2% lactate). SD medium (0.67% yeast nitrogen base without amino acids, 2% glucose) or SLac medium (0.67% yeast nitrogen base without amino acids, 2% lactate) supplemented with 25 μg/ml of the required amino acids were used to grow strains carrying plasmids. When necessary, 200 μg/ml geneticin (G418) was added to the media for selection of mutant strains.

### Buffers and Solutions

The detailed composition of buffers and solutions used in this study is described in Table S3.

### Cell Fractionation

Mitochondrial fractions were prepared essentially as described [17]. Yeast cells were grown on YPLac for 24 h, collected by centrifugation, washed with water, and resuspended in 10 ml of Tris-DTT buffer, followed by incubation 10 min at 30 °C. Cells were pelleted by centrifugation, resuspended in 10 ml zymolyase buffer, and incubated for 1 h at 30 °C with gentle shaking. The resulting spheroplasts were then pelleted, resuspended in 1 ml of homogenization buffer, and broken using 15 strokes with a cold pressure homogenizer. Cell debris and nuclei were removed by centrifugation for 5 min at 4000 rpm (4 °C). The supernatant was centrifuged at 12,000 rpm for 10 min (4 °C) to separate the mitochondrial fraction (pellet) from the cytoplasm fraction (supernatant). The cytoplasm fraction (CF) was stored directly at – 70 °C, while the mitochondrial fraction (MT) was first resuspended in SH buffer prior to storage at – 70 °C. Protein concentration was determined using the Pierce BCA Assay Kit (Thermo Scientific).

### Tau Detection in Mitochondrial Compartments

Suspensions containing 5 μg of crude mitochondria were diluted 1:10 with either isotonic medium (to maintain intact mitochondria) or hypotonic medium (to generate mitoplasts) and incubated for 30 min on ice. When indicated, samples were treated with 50 μg/ml of proteinase K (PK) for 20 min on ice. Reactions were stopped by adding 400 µl SH KCl buffer and 1 mM PMSF. Triton-X 100 (0.2%) was added for complete solubilization of mitoplasts.

### Western Blot Analysis

Yeast cells were collected by centrifugation and resuspended in Rodel mix supplemented with 4% protease inhibitor cocktail. Cells were then precipitated with 85% trichloroacetic acid (TCA) and washed once with 1 ml ice-cold acetone. Cells were then resuspended in modified 1 × Laemmli lysis buffer, and protein concentration was determined using the Pierce-BCA Assay (Thermo Scientific). Samples were loaded on a 12% SDS-PAGE Tris/HCl gel, transferred onto PVDF membranes (Millipore), and blocked with 10% skim milk in 1% TBS-Tween. Membranes were probed with the corresponding antibodies listed in Table S4, according with the suppliers’ recommendations. Reactive proteins were visualized using the Immobilon Western Chemiluminescent HRP Substrate (Millipore) and images were digitized using the C-DiGit Blot Scanner (LI-COR). Image analysis was performed using Image Studio Digits software, version 5.2.

### Quantification of Mitochondrial Morphotypes

Wild type cells carrying either pVT100-tau or the empty vector were grown overnight (mid-log phase) in SD medium, collected, adjusted to OD_600_ 0.2, and allowed them to reach OD_600_ 0.5. Cells were observed under confocal microscopy. Mitochondrial morphotypes were quantified by analyzing 100 cells per experiment, and the percentage of cells displaying either filamentous or fragmented mitochondrial morphology was determined.

### Oxygen Consumption

Cells grown for 24 h at 30 °C on YPD medium were collected by centrifugation, washed, and starved in water for 24 h. After starvation, cells were pelleted and oxygen consumption was measured by adding 25 mg of wet cell mass to a chamber containing 5 ml of 10 mM MES-TEA buffer and 20 mM of glucose or ethanol. To uncouple oxidative phosphorylation (OXPHOS), 4 μM of carbonyl cyanide 3-chlorophenylhydrazone (CCCP) was added stepwise. Cell suspension was maintained under constant stirring at 30 °C. The rate of consumption was determined using a Clark-type oxygen electrode connected to a Steren multimeter (model MUL-600) and a YSI Oxygen Monitor 5300 (Yellow Springs Instrument). Data were analyzed using the MUL-600 software.

### Membrane Potential Determination

Mitochondrial membrane potential was determined using the fluorescent dye Rhodamine 123 (Invitrogen), following a previously described protocol with minor modifications [18]. Cells were grown in SD medium for different times, collected by centrifugation, washed with water, and adjusted to an OD_600_ 0.08. Cells were stained with 62.5 nM Rhodamine 123 for 10 min at room temperature (RT) in the dark then washed twice with water. Mitochondrial fluorescence intensity was quantified by flow cytometry. Net fluorescence was calculated by subtracting the fluorescence intensity of unstained control cells from that of stained cells. To account for cell size variation, the resulting fluorescence value was normalized by the forward scatter (FSC) signal.

### Cytochrome Quantification

Mitochondria (1.5 mg/ml) were purified from cells grown in YPLac medium for 24 h. A volume of 500 µl of mitochondria suspension was incubated with either 1 mg potassium ferricyanide (oxidized state) or 1 mg sodium dithionite (reduced state) at 25 °C with shaking. Absorbance spectra were immediately recorded using a Clarity VF UV–visible Olis spectrophotometer over a wavelength range of 400–700 nm. The final difference spectrum was obtained by subtracting the reduced-state spectrum from the oxidized-state spectrum. Cytochrome content was calculated using the following molar extinction coefficients: for cytochromes *a* + *a*3, *ε* 604–630 = 24 mM^−1^cm^−1^; for cytochrome *b*, *ε* 563–577 = 28 mM^−1^cm^−1^; and for cytochromes *c* + *c*1, *ε* 553–539 = 19.1 mM^−1^cm^−1^). Cytochrome concentrations were determined by dividing the absorbance change (Δ*A*) by the corresponding molar extinction coefficient, normalized to protein content.

### Viability Quantification

Cells grown in SD or SLac for various times were collected by centrifugation, adjusted to OD_600_ 0.8, and washed with 10 mM HEPES buffer. Cells were then resuspended in GH buffer containing 400 μM FUN1 dye (Invitrogen) and incubated in the dark for 15 min at 30 °C with gentle shaking. After incubation, cells were washed with 1 × PBS and observed under epifluorescence microscopy. For each assay, 500 cells were counted, and the percentages of red- and green-fluorescing cells were determined. Cells heated at 96 °C for 10 min served as a control for cell death.

### Mitophagy Assays

To evaluate mitophagy by nitrogen starvation or during stationary phase, we followed previously described protocols [19]. Briefly, strains expressing the mitochondrial protein Idh1 fused to GFP were grown to mid-log phase in YPD or SD medium at 30 °C. For nitrogen starvation, cells were transferred to YPLac and grown for 14 h until reaching an OD_600_ of 2.0. Cells were washed twice with water and transferred to SD lacking nitrogen, followed by incubation for various times before harvesting. To induce mitophagy in stationary phase, cells were grown in YPD for 14 h and shifted to YPLac. Aliquots were taken at 24, 48, and 72 h. Protein extracts were prepared and subjected to western blot.

### Fluorescence Microscopy

Cells expressing fluorescence dyes and/or fluorescent-tagged proteins were visualized under epifluorescence microscopy with an Olympus BX51 microscope or confocal microscopy with a Zeiss LSM800 spectral system. Images were analyzed using the Ocular V2.0 software for epifluorescence images and Zen2 (Carl Zeiss) software for confocal images.

### Flow Cytometry

Cells were grown to OD_600_ of 0.5, and fluorescence intensity was measured using the Attune Acoustic Focusing Cytometer (Applied Biosystems). Fluorescence data were collected from 50,000 cells, and analysis was performed with the Attune Cytometric Software v2.1.

### Statistical Analysis

Statistical analysis was performed on data obtained from at least three independent experiments using GraphPad Prism software Version 9. Comparisons between groups were conducted using parametric Student’s *t*-tests. Statistical significance was set at *p* < 0.05. Asterisks denote significance levels as follows: *p* < 0.05 (*), *p* < 0.01 (**), and *p* < 0.001 (***). In all graphs, error bars represent the standard deviation.

## Results

### Tau Expression and Phosphorylation in Yeast

**Fig. 1:**
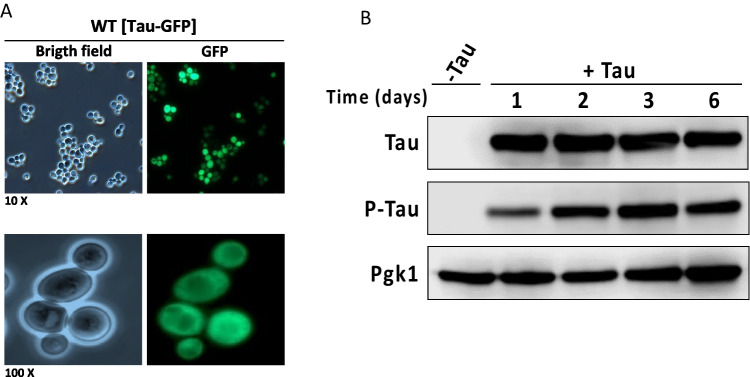
Human 0N3R tau is widely expressed and phosphorylated in *S. cerevisiae* cells. **A** The 0N3R ORF tagged with GFP was expressed under the control of the constitutive *ADH1* promoter using the pVT100 vector. Cells were grown in YPD medium for 24 h at 30 °C and imaged by epifluorescence microscopy. **B** Detection of tau using total tau and phospho-tau antibodies. Total protein extracts were prepared from cells transformed with either the pVT100-tau construct, which carries untagged tau, or the empty vector and grown in SD medium for different times. Western blot analysis was performed using the Tau12 antibody to detect total tau and a phospho-specific antibody recognizing tau phosphorylated at Ser199/202. Pgk1, detected with an anti-Pgk1 antibody, was used as a loading control

The use of yeast as a model system provides a simplified yet effective platform for studying physiological effects of tau expression and post-translational modifications, such as phosphorylation. In this work we constitutively expressed the full-length human isoform 0N3R and a carboxyl-end GFP tagged variant in order to detect its cellular distribution, its phosphorylation, and its consequences on mitochondrial dynamics and metabolism. Our observations revealed that this form of tau exhibits widespread expression in exponentially growing cells (Fig. 1A). Then, we monitored the expression of untagged tau over time using western blot analysis and assessed whether tau phosphorylation occurred at Ser199/202, which are key residues implicated in tau aggregation in Alzheimer’s disease in all six isoforms [20, 21]. Due to the constitutive nature of the promoter, we observed consistently high levels of tau protein over a 6-day period. Notably, a significant proportion of tau was found phosphorylated at Ser199/202 (Fig. 1B), indicating that the 0N3R isoform is actively modified in yeast cells.

### A Fraction of Tau Is Localized in Mitochondria

**Fig. 2:**
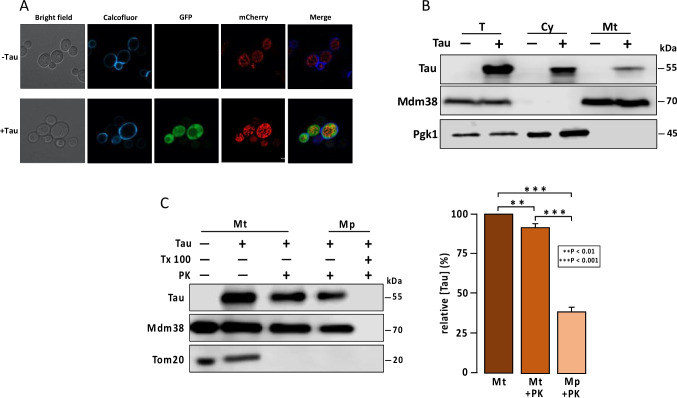
A fraction of tau is detected in mitochondrial compartments. **A** Representative confocal microscopy images showing colocalization of tau-GFP with mitochondria labeled with mt-mCherry. Cells carrying pVT100-tau or empty vector with pYX122-mt-mCherry were grown until mid-log phase in SD medium. The cell wall was stained with Calcofluor. Images were acquired using confocal microscopy. **B** Detection of tau in total, cytosolic, and mitochondrial fractionations. Cells carrying pVT100-tau, expressing untagged tau, or empty vector were grown in SD medium. Tau was detected using the Tau12 antibody. Pgk1 and Mdm38 were used as markers for the cytosolic and mitochondrial fraction, respectively. **C** Detection of untagged tau in mitochondrial compartments. Mitochondria and mitoplast obtained from cells carrying pVT100-tau or empty vector were treated with proteinase K (PK) and triton X100 (Tx 100) as indicated. Tom20 was used as a marker of the outer mitochondrial membrane and Mdm38 as a marker for the mitochondrial matrix. Densitometric analysis from three independent experiments of tau detection in mitochondria (with or without PK treatment) and mitoplast treated with PK, relative to total mitochondrial tau (100%) is shown. T, total extract,; Cy, cytoplasmic fraction; Mt, mitochondrial fraction; Mp, mitoplasts

We next investigated whether tau could localize to mitochondria. Using confocal microscopy, we observed that a fraction of tau tagged with GFP colocalized with an mCherry construct targeted to mitochondria via an import sequence (Fig. 2A). To further examine this tau incorporation, we analyzed the distribution of untagged tau by western blot following cell fractionation. The results showed that while a portion of tau was found in the cytoplasmic fraction, a significant amount was also detected in the mitochondrial fraction (Fig. 2B). To pinpoint the specific mitochondrial compartment where tau resides, we prepared protein extracts from intact mitochondria and from mitoplasts, with or without treatment with proteinase K (PK). Our findings revealed that a substantial proportion of tau was protected from PK digestion in isolated mitochondria and a fraction of this protein was also PK-resistant in mitoplasts (Fig. 2C). These results strongly suggest that tau is incorporated into both the mitochondrial intermembrane space and the mitochondrial matrix.

### Mitochondrial Import of Tau Depends on the Presence of Hsp104 Disaggregase and the Bichaperon Complex Ssa1/Ydj1

**Fig. 3:**
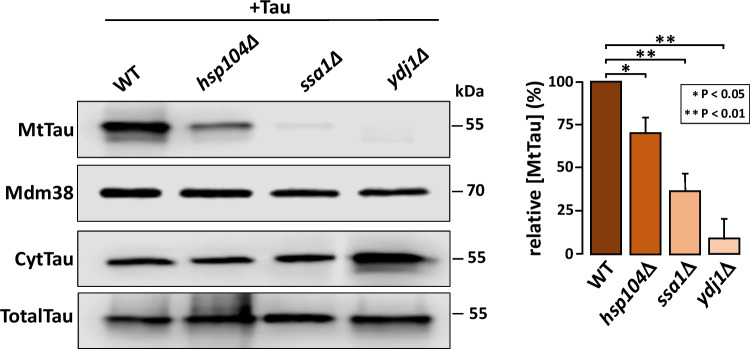
The chaperon complex Hsp104, Ssa1, and Ydj1 promotes tau mitochondrial translocation. Representative immunoblot showing tau protein detected with Tau12 antibody. Yeast strains, carrying pVT100-Tau, expressing untagged tau, were grown in SD medium until mid-log phase, incubated 24 h in YPLac, and fractionated to obtain the mitochondrial fraction. Densitometric analysis of mitochondrial tau (MtTau) from three independent experiments is shown. Values were first normalized to the mitochondrial protein Mdm38 and then to the WT strain. Tau detected in the cytoplasmic fraction (CytTau) and in total cellular extracts (TotalTau) are shown. Bar graphs represent means ± standard deviation

Mitochondrial uptake of aggregating proteins requires a chaperone activity composed of the tripartite Hsp104/Ssa1/Ydj1 complex, where Hsp104 acts as a disaggregase [22, 23]. Therefore, we tested whether this complex was involved in the mitochondrial import of the tau protein. To this end, we fractionated mitochondria and detected tau by western blot. Mitochondria from the single mutants *hsp104Δ*, *ssa1Δ*, and *ydj1Δ* exhibited reduced tau uptake compared to the wild-type strain (Fig. 3). Interestingly, we observed that in mutants lacking Ssa1 or Ydj1 (proteins that are thought to act together as a bichaperone complex), mitochondrial import of tau was significantly impaired. Tau expression in these strains was not altered, as indicated by comparable levels detected in both, total cell extracts and the cytoplasmic fraction. This result suggests that tau must first be solubilized to be effectively transported into mitochondria.

### Tau Expression Induces Mitochondrial Fragmentation and Impairment of Oxygen Consumption

**Fig. 4:**
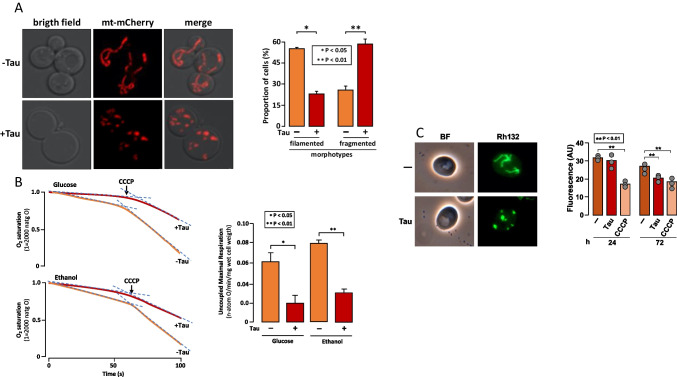
Tau induces mitochondrial fragmentation and defective respiration.** A** Representative images of mitochondria labeled with mt-mCherry. WT cells carrying pVT100-tau, expressing untagged tau, or the empty vector were grown in SD medium to mid-log phase and imaged using confocal microscopy. The proportion of mitochondrial morphotypes was determined by counting 100 cells. Bar graphs represent the mean ± standard deviation. **B** Oxygen consumption rate in whole yeast cells carrying pVT100-tau, expressing untagged tau (red line) or empty vector (orange line). Cells were grown on YPD for 24 h. After 24h starvation in water, wet cells were resuspended in MES-TEA buffer pH 6.0, and the oxygen consumption was measured using glucose or ethanol as substrates. CCCP was added to induce maximum oxygen consumption by uncoupling OXPHOS. Densitometric analysis from four independent experiments assessing maximum oxygen consumption is shown. Bars represent the average slope values under CCCP treatment ± standard deviation. **C** Mitochondrial membrane potential. Cells expressing or not expressing tau were incubated with 62.5 nM Rhodamine 123, as described in the “Materials and methods” section. A representative epifluorescence image is shown. Densitometric analysis was performed in three independent experiments. Data represent the mean, with error bars indicating the standard deviation

To assess the consequences of untagged tau expression on mitochondrial morphology, we studied cells labeled with a mitochondrial-targeted mCherry protein using fluorescence microscopy. In exponentially growing cells, where a significant fraction of tau is phosphorylated, we observed that the mitochondrial network was highly fragmented in the presence of tau, compared to the predominantly filamentous mitochondria seen in wild-type cells (Fig. 4A). The proportion of cells with fragmented mitochondria increased from 20% in the absence of tau to 60% when tau was expressed. Conversely, the percentage of cells with filamentous mitochondria decreased from approximately 60% without tau to 20% with tau expression (Fig. 4A), demonstrating that tau expression has a pronounced impact on mitochondrial dynamics, promoting fragmentation over the maintenance of a filamentous network. Due to the observed morphological changes, we next evaluated whether tau protein affects mitochondrial respiration in intact cells. To this end, we monitored oxygen consumption using glucose or ethanol as respiratory substrates. We observed that cells expressing tau exhibited a reduced rate of oxygen consumption with both glucose and ethanol (Fig. 4B). We then examined the effect of the oxidative phosphorylation uncoupler CCCP on the oxygen consumption rate to evaluate the maximal respiratory capacity. In the presence of tau, CCCP induced a weaker acceleration of oxygen consumption compared to cells not expressing tau (Fig. 4B). In addition, we investigated whether tau induces changes in mitochondrial membrane potential. While no effect was detected in exponentially growing cells, a moderate but significant drop in membrane potential was observed in cells that had reached the stationary phase (Fig. 4C). In contrast, the cytochrome content (*c* + *c*1, *b*, and *a* + *a*3) remained unchanged in both exponentially growing and stationary phase cells (Fig. S2). We concluded that tau may impair mitochondrial function by affecting both the respiration rate and the proton gradient across the inner mitochondrial membrane. Finally, we assessed whether tau-induced mitochondrial dysfunction impacts cell viability, but only a moderate reduction in viability was observed in stationary phase cells grown in either glucose or lactate (Fig. S3).

### Tau Induces the Yeast Retrograde Response

**Fig. 5:**
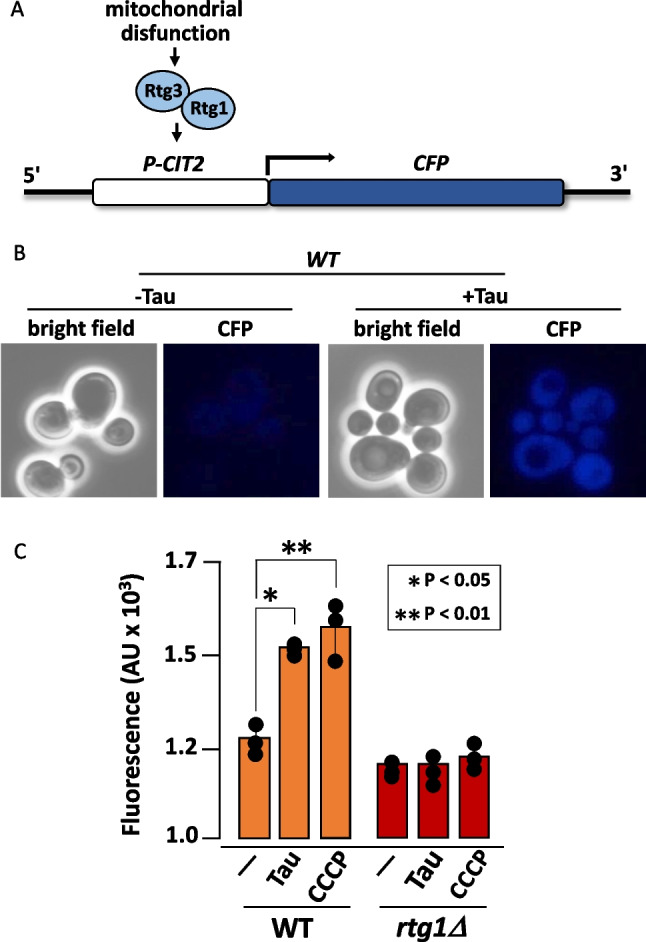
Tau expression activates the mitochondrial retrograde response.** A** Schematic representation of the gene encoding the cyan fluorescent protein (CFP) under the control of the *CIT2* promoter. Mitochondrial dysfunction regulates the activity of transcription factors Rtg1 and Rtg3. **B** Representative image of CFP signal detected by epifluorescence microscopy. Cells transformed with either the empty pVT100 or pVT100-tau were grown in SD medium to mid-log phase and photographed. **C** Quantitative analysis of CFP fluorescence intensity measured by flow cytometry. Treatment with the uncoupler CCCP was used as positive control for induction of the retrograde response

Mitochondrial dysfunction induces a nuclear transcriptional response known as the retrograde pathway. This pathway requires, among other proteins, the participation of Rtg1 and Rtg3, which act as transcription factors on a variety of promoters. *CIT2*, which encodes a peroxisomal citrate synthase, has been identified as a target gene of Rtg1/Rtg3 under activation of the retrograde pathway [24]. To monitor retrograde pathway activation, we fused the gene encoding CFP (cyan fluorescent protein) to the *CIT2* promoter. In wild-type cells expressing tau, we observed a two-fold increase in fluorescence compared to cells carrying the empty vector (Fig. 5). The tau-dependent induction of *CIT2* expression was comparable to the increase triggered by CCCP treatment, a classical inducer of the retrograde response. To confirm that *CIT2/CFP* induction occurred via the retrograde pathway, we expressed tau in an *rtg1Δ* mutant. In this strain, the *CIT2* promoter was not activated by either tau expression or CCCP treatment (Fig. 5). Thus, mitochondrial dysfunction caused by tau expression activates the retrograde signaling pathway, leading to transcriptional reprogramming in the nucleus.

### Tau Expression Increases Mitochondrial Clearance Through Mitophagy

**Fig. 6:**
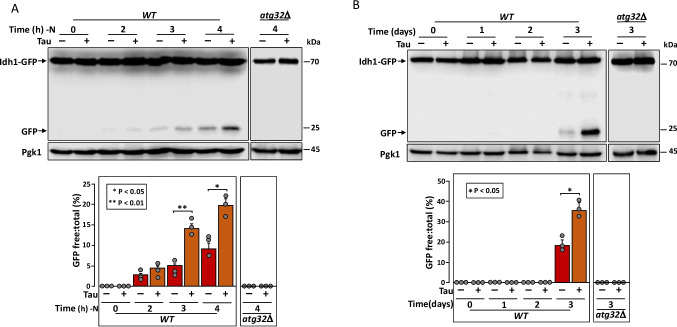
Tau expression increases mitophagy.** A** Representative immunoblot showing mitophagy induction by the detection of free GFP at various time points during nitrogen starvation (–N). Cells expressing the Idh1-GFP protein were transformed with either pVT100 or pVT100-tau, grown in SD medium, transferred to YPLac for 14 h, and then shifted to SD medium lacking nitrogen. **B** Representative immunoblot showing mitophagy during the stationary phase. Cells expressing the Idh1-GFP were transformed with pVT100 or pVT100-tau, grown in SD medium, and transferred to YPLac for the indicated times. In both panels, the ratio of free GFP to total GFP (Idh1-GFP) was quantified by densitometry from three independent experiments. Data represent the mean free:total GFP ratio, with error bars indicating standard deviation. Pgk1 was used as loading control. An *atg32Δ* strain, which lacks the mitophagy pathway, was used as negative control

Due to the effects of tau expression on yeast mitochondrial morphology and metabolism, we explored whether these alterations impact the mitophagy process induced by nitrogen starvation or during stationary phase. To this end, we constitutively expressed tau in yeast cells carrying Idh1 (isocitrate dehydrogenase) fused to GFP. During mitophagy, Idh1-GFP is delivered to the vacuole, where it is processed to release free GFP, which can be detected by western blotting as an indicator of mitochondrial degradation [25]. This pathway depends on the interaction between the mitochondrial receptor Atg32 and a protein complex located in the phagophore. This interaction facilitates the recruitment of mitochondria to the autophagic machinery and their subsequent import into the vacuole for complete degradation. The absence of Atg32 completely blocks mitophagy [26]. After 2 h of nitrogen starvation, mitochondrial degradation in tau-expressing cells was similar to that observed in cells carrying the empty vector. However, at 3 and 4 h, mitochondrial degradation increased progressively, and in cells expressing tau, the level of degradation was significantly greater than in control cells (Fig. 6A). A similar pattern was observed when mitophagy was induced by culturing cells in a medium containing a non-fermentable carbon source and allowing them to reach stationary phase. After 3 and 4 days, mitochondrial degradation was again significantly higher in cells expressing tau compared to cells carrying vector alone (Fig. 6B). These time-course experiments indicate that tau expression enhances mitochondrial clearance through mitophagy.

### Tau-Induced Mitophagy Requires the Retrograde Pathway

**Fig. 7:**
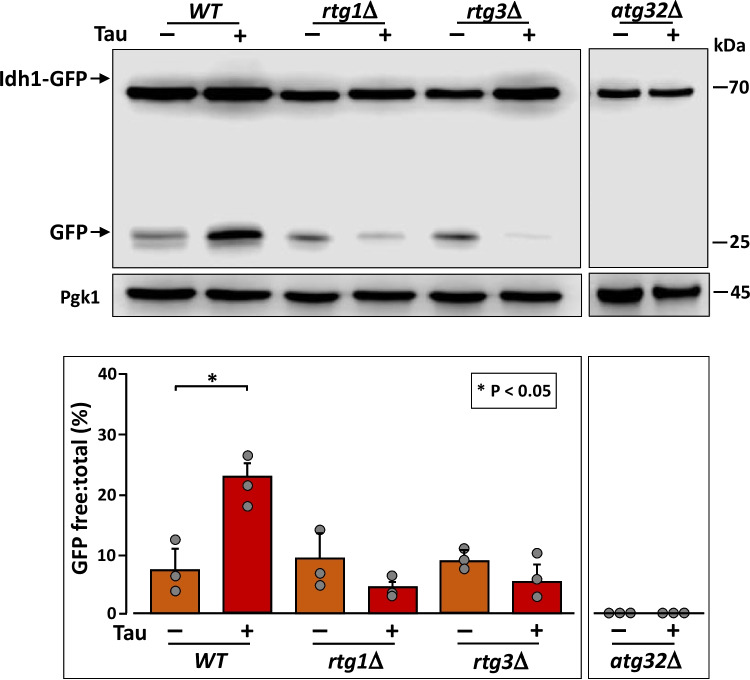
Tau induction of mitophagy depends on the retrograde response. Representative immunoblot showing mitophagy during stationary phase, assessed by detection of free GFP. Cells expressing the Idh1-GFP protein were transformed with pVT100 or pVT100-tau, grown in SD medium, and transferred to YPLac for 3 days. Quantification of free GFP and total GFP (Idh1-GFP) was performed by densitometry from three independent experiments. The mean of free:total GFP ratio is shown, with error bars representing standard deviation. Pgk1 protein was used as loading control

Since the expression of tau induces the retrograde response and enhances mitophagy, we asked whether these two processes are connected in cells expressing tau. To address this, we monitored mitophagy in mutant cells lacking *RTG1* or *RTG3*. We observed that the increased level of mitophagy seen in wild-type cells expressing tau was not present in cells lacking components of the retrograde response (Fig. 7). Moreover, tau expression significantly reduced the basal level of mitophagy in the *rtg1Δ* and *rtg3Δ* mutants. These findings suggest that, in order to promote increased mitochondrial degradation, tau requires an active retrograde signaling pathway.

## Discussion

In this study, we utilized *Saccharomyces cerevisiae* as a simplified eukaryotic model to explore the cellular effects of human tau expression, with a particular focus on its impact on mitochondrial function to dissect some of the mechanisms involved in tau toxicity. Our results demonstrate that the full-length 0N3R isoform of human tau can be widely expressed in exponentially growing cells, consistent with previous studies showing that tau exhibits diffuse cytoplasmic localization in yeast [27]. Further analysis revealed that tau remained phosphorylated at Ser199 and Ser202, residues known to be critical in the development of tau pathology in Alzheimer’s disease [20, 21, 28]. The Ser 199/202 residues are substrates of different kinases such as GSK3B and CdK5 [29]. Interestingly, yeast expresses orthologs of the same kinases, Pho85 and Mds1, respectively, capable of phosphorylating tau at the same Alzheimers-related residues, as has been previously demonstrated [30, 31].

Our data show that 0N3R isoform of tau is not only present in the cytoplasm but a fraction was also localized within internal mitochondrial yeast compartments, with a significant proportion reaching the matrix. This internal localization implies that tau may translocate across mitochondrial membranes, which correlates in part with the finding that 2N4R-tau is distributed at the outer mitochondrial membrane and in the intermembrane space in HeLa cells [32]. Other reports have documented the presence of tau into synaptic mitochondria associated with mitochondrial dysfunction in the hippocampus from aged mice [7] and in a mouse model of Alzheimer’s disease [6] without describing the mechanism involved in tau transport. Here we report for the first time that efficient mitochondrial import of tau protein in yeast requires the action of a specialized chaperone complex composed of Hsp104, Ssa1, and Ydj1. These chaperones are known for their roles in disaggregating and solubilizing misfolded proteins, with Ssa1 and Ydj1 specifically facilitating early aggregates handling, and Hsp104 functioning as disaggregase [22, 33]. Deletion of any of these chaperones reduced mitochondrial tau levels, suggesting that tau must be partially unfolded to be imported via the so-called MAGIC (mitochondria as guardian in cytosol) pathway [23]. These findings suggest that the Hsp104/Ssa1/Ydj1 chaperone machinery plays a critical role in preparing pathological proteins such as tau for mitochondrial import and subsequent degradation. Interestingly, yeast Ydj1 is an ortholog of the human chaperone DnaJA1 which has been implicated in the prevention of tau phosphorylation and its consequent aggregation in neuronal models [34].

In our study, we found that expression of 0N3R-tau in yeast cells led to a significant increase in mitochondrial fragmentation. This supports previous observations that pathogenic forms of tau disrupt mitochondrial dynamics in neuronal models, often through interactions with fission/fusion machinery or cytoskeletal elements that anchor mitochondria [35, 36]. This fragmentation may have downstream consequences for organelle function. We observed that tau expression significantly reduced oxygen consumption in the presence of both glucose and ethanol, suggesting impaired electron transport chain activity. Additionally, a moderate but statistically significant drop in membrane potential was observed in stationary-phase cells expressing tau. This temporal effect suggests that tau toxicity may become more pronounced as cells encounter metabolic stress, consistent with observations in aging or neurodegenerative models where energy demand exceeds mitochondrial capacity [6, 37]. Interestingly, recent studies have shown that Aβ oligomers disrupt the conductance and ion selectivity of voltage-dependent anion-selective channel 1 (VDAC1), leading to impaired mitochondrial respiration and reduced cell viability [38]. Together, these findings highlight mitochondrial dysfunction as a shared feature in tau- and Aβ-related neurotoxicity.

Mitochondrial dysfunction is increasingly recognized as a central player in the pathogenesis of neurodegenerative diseases. One of the key cellular responses to compromised mitochondrial function in yeast is the activation of the retrograde signaling pathway, a conserved communication mechanism through which mitochondria signal their functional status to the nucleus [39, 40]. This pathway involves the transcription factors Rtg1 and Rtg3, which translocate to the nucleus upon mitochondrial stress and regulate genes associated with metabolic adaptation [41]. In this study, we detected that 0N3R-tau expression activates the retrograde response as a consequence of its detrimental effects on mitochondrial function. These findings reinforce the notion that mitochondrial quality control and retrograde signaling are tightly coupled and that disruption of mitochondrial homeostasis by pathological proteins such as tau can lead to broader cellular reprogramming, a concept further supported by evidence showing that the activation of the mitochondrial unfolded protein response (UPR^mt^) exerts a protective role against Aβ proteotoxicity in mammalian systems [42, 43], highlighting conserved mitochondria-to-nucleus communication pathways present in Alzheimer’s disease.

Consistent with previous studies [44, 45], our results showed that 0N3R-tau expression enhances mitochondrial clearance via mitophagy under nutrient stress. This enhancement of mitophagy may represent a compensatory cellular response to tau-induced mitochondrial dysfunction. Contrasting studies have reported that pathological tau impairs mitophagy in mammalian and *C. elegans* systems, through increasing the mitochondrial potential and inhibition of Parkin recruitment [46, 47]. Since yeast lacks the PINK1-Parkin mitophagy pathway present in metazoans, this discrepancy may arise from species-specific differences in mitochondrial quality control mechanisms and/or differences in the tau isoforms used across studies. Interestingly, we found that disruption of the retrograde signaling pathway, via deletion of either *RTG1* or *RTG3*, abolished the tau-induced increase in mitophagy, suggesting that the retrograde signaling acts upstream of the tau-induced mitophagy pathway. This is consistent with studies showing that mitochondrial signaling can regulate autophagic responses via transcriptional reprogramming [40].

The expression of tau protein in yeast provides a powerful platform for understanding the cellular and molecular effects of its toxicity. It highlights conserved mechanisms, such as mitochondrial dysfunction, proteostasis imbalance, and autophagy impairment, that are relevant to tauopathies in humans. This system continues to be instrumental in identifying potential therapeutic targets for mitigating tau-induced cellular damage.

### Acknowledgements

We acknowledge technical assistance of the staff at Instituto de Fisiología Celular: Minerva Mora (Unidad de Biología Molecular); Ximena Lezama, José Antonio Torres, Juan Barbosa and Ivette Rosas (Unidad de Computo); Manuel Ortínez and Aurey Galván (Taller de Mantenimiento); Ruth Rincón and Abraham Rosas (Unidad de Imagenología); Ma. Teresa Lara Ortíz (Colección de Cepas de Levadura).

**Author Contribution**

Conception and design of this study: YC, CA, SF, RC; experiments conduction, data collection, and analysis: YC, LK, HR, NSS, MGC, LO; manuscript first draft: YC, RC; final writing and approval of the manuscript: all authors.

#### Funding

CONAHCyT (SECIHTI) Grant No. CF58550 to RC, CA, SF; PAPIIT, DGAPA, UNAM Grant No. IN207223 to RC; YC-C, received a fellowship from CONAHCyT (CVU: 1,007,708) and from grant CF58550.

## Supplementary Information

Below is the link to the electronic supplementary material.

Supplementary file1 (DOCX 1.98 MB)

Supplementary file2 (DOCX 22.0 KB)

Supplementary file3 (DOCX 23.1 MB)

## Data Availability

No datasets were generated or analyzed during the current study.
