# Supplementary material for: Tau Protein Disrupts Mitochondrial Homeostasis in a Yeast Model: Implications for Alzheimer’s Disease
Source: Mol Neurobiol. 2025 Aug 8;62(12):16460–71. doi: 10.1007/s12035-025-05255-z (PMC12559152; doi:10.1007/s12035-025-05255-z)
Supplement: Supplementary file 1 — Supplementary file1 (DOCX 1.98 MB) [file 12035_2025_5255_MOESM1_ESM.docx]

Supplementary Material:

**Tau protein disrupts mitochondrial homeostasis in a yeast model: implications for Alzheimer’s disease**

Yaisa Castillo-Casaña^1^, Laura Kawasaki^1^, Clorinda Arias^2^, Hilario Ruelas-Ramírez^3^, Soledad Funes^3^, Norma Silvia Sánchez^3^, María Guadalupe Códiz-Huerta^4^, Laura Ongay-Larios^4^ and Roberto Coria^1^*.

**Figure S1**.

The six major isoforms of tau protein generated by alternative splicing of the MAPT gene. The 0N3R isoform is the shortest.

**Figure S2.**

**Mitochondrial cytochrome content.** Cytochrome spectral traces recorded from isolated mitochondria as indicated in Materials and Methods. The final difference spectrum was obtained by subtracting the reduced-state spectrum from the oxidized-state spectrum. Cytochrome concentrations were determined by dividing the absorbance change (ΔA) by the corresponding molar extinction coefficient, normalized to protein content as indicated in Materials and Methods.

**Figure S3**.

**Cell viability determination2.** Cells grown for the indicated times were stained with 400 uM FUN1 dye as indicated in the Materials and Methods section. Representative epifluorescence microscopy images are shown. 500 cells were counted in three independent experiments and the proportion of green and red cells was determined. Viable cells are visualized in red fluorescence, while death cells are visualized in green. (*) p<0.05.
