# Supplementary material for: Tau Protein Disrupts Mitochondrial Homeostasis in a Yeast Model: Implications for Alzheimer’s Disease
Source: Mol Neurobiol. 2025 Aug 8;62(12):16460–71. doi: 10.1007/s12035-025-05255-z (PMC12559152; doi:10.1007/s12035-025-05255-z)
Supplement: Supplementary file 2 — Supplementary file2 (DOCX 22.0 KB) [file 12035_2025_5255_MOESM2_ESM.docx]

Supplementary Material:

**Tau protein disrupts mitochondrial homeostasis in a yeast model: implications for Alzheimer’s disease**

Yaisa Castillo-Casaña^1^, Laura Kawasaki^1^, Clorinda Arias^2^, Hilario Ruelas-Ramírez^3^, Soledad Funes^3^, Norma Silvia Sánchez^3^, María Guadalupe Códiz-Huerta^4^, Laura Ongay-Larios^4^ and Roberto Coria^1^*.

**Table S1. Yeast strains used in this work**.

| Strain | Genotype | Source |
| --- | --- | --- |
| BY4741 WT | MATa *his3Δ1 leu2Δ0 met15Δ0 ura3Δ0* | Euroscarf |
| BY4742 WT | MAT*α his3Δ1 leu2Δ0 lys2Δ0 ura3Δ0* | Euroscarf |
| *rtg1Δ* (BY4742) | MAT*α his3Δ1 leu2Δ0 lys2Δ0 ura3Δ0* *rtg1:KanMX4* | Euroscarf |
| *hsp104Δ* (BY4742) | MAT*α his3Δ1 leu2Δ0 lys2Δ0 ura3Δ0* *hsp104:KanMX4* | Euroscarf |
| *ssa1Δ* (BY4742) | MAT*α his3Δ1 leu2Δ0 lys2Δ0 ura3Δ0* *ssa1:KanMX4* | Euroscarf |
| *ydj1Δ* (BY4742) | MAT*α his3Δ1 leu2Δ0 lys2Δ0 ura3Δ0* *ydj1:KanMX4* | Euroscarf |
| *WT Idh1-GFP* | *MATa his3Δ1 leu2Δ0 met15Δ0 IDH1-GFP::HIS3 ura3Δ0* | GFP-tagged collection |
| *atg32Δ Idh1-GFP* | *MATa his3Δ1 leu2Δ0 met15Δ0 IDH1-GFP::HIS3 ura3Δ0 atg32::kanMX4* | García-Chávez, et al, 2024 |
| *rtg1Δ Idh1-GFP* | *MATa his3Δ1 leu2Δ0 met15Δ0 IDH1-GFP::HIS3 ura3Δ0 rtg1::KanMX4* | This work |
| *rtg3Δ Idh1-GFP* | *MATa his3Δ1 leu2Δ0 met15Δ0 IDH1-GFP::HIS3 ura3Δ0 rtg3::KanMX4* | This work |

**Table S2. List of oligonucleotides used in this work.**

| Name | Sequence (5'-3') | starting ORF position | Restriction sites |
| --- | --- | --- | --- |
| Tauf | AAGCTTGGTACCATGGCTGAG | +1 | HindIII, KpnI |
| Taur | GAGCTCTAGATTACAAACCCTGCTTGGCCAGGGA | +1036 | SacI, XbaI |
| cit2promf | GAGCTCGTTTATCACTGCGATATTGGA | -896 | SacI |
| cit2promr | CCCGGGTGAATTTAGATAAGGAACTGT | +4 | XmaI |
| rtg1ΔF | AACACTAGATAGTGAACCAAAAGAAAGCACAACACCAAACccagctgaagcttcgtacgc | +2307 |  |
| rtg1Δr | GGTTATCACAACATAGCAATAGTGAGAGTCAGAAGTACTTtcgatgaattcgagctcgtt | +3802 |  |
| rtg3ΔF | ATTTTTTGTCAGGCGAACCTACTTCTTAAATAAGTGAAGAccagctgaagcttcgtacgc | +2307 |  |
| rtg3Δr | TTTTTCAAATTTAATTTTTTCCCGCTAATAAGACCATAAAtcgatgaattcgagctcgtt | +3802 |  |

Red bases indicate the positions of restriction sites. Hybrid oligonucleotides: lowercase bases correspond to the G418 cassette.

**Table S3. List of growth media, buffers and solutions used in this work**

| Solution | Composition |
| --- | --- |
| YPD medium | glucose 2%, yeast extract 1%, peptone 2% and agar 2% for solid medium only |
| SD medium | glucose 2%, yeast nitrogen base without amino acids 0.67% |
| YPLac medium | lactate 2%, yeast extract 1%, peptone 2% |
| SD-N medium | glucose 2%, yeast nitrogen base without nitrogen and amino acids 0.19% |
| Tris-DTT | 100 mM Tris and 10 mM DTT |
| Zymolyase buffer | 1.2M Sorbitol, 10 mM KH2PO4, Zymolyase 5 mg/g wet weight |
| homogenization buffer | 0.6M Sorbitol, 10mM Tris pH 7.4, 1mM EDTA, 0.2% BSA and 1mM phenylmethylsulfonyl fluoride (PMSF) |
| sh buffer | 0.6 M Sorbitol, 20 mM Hepes |
| Isotonic medium | 20 mM HEPES pH 7.4, 0.6 M Sorbitol |
| hypotonic medium | HEPES 20mM pH 7.4 |
| SH kcl buffer | 20 mM HEPES pH 7.4, 0.6 M Sorbitol, 80 mM KCl |
| 1x Leammli buffer | SDS (2%), Glycerol (10%), β-mercaptoethanol (5%), Bromophenol blue (0.002%) and Tris-HCl pH 6.8 (62.5%) |
| Rodel mix | NaOH (18.5%), β-mercaptoethanol (7.4%), PMSF (10%) |
| modified 1x Leammli buffer | SDS (2%) and Tris-HCl pH 6.8 (62.5%) |
| tbs-tween | 0.1% Tween 20, 150mM NaCl, 50 mM Tris, pH 7.5 |
| GH buffer | 10mM Hepes pH 7.2, Glucose 2% |
| PBS1X | NaCl 137mM, KCl 2.7mM, Na_2_HPO_4_ 10mM, KH_2_PO_4_ 1.8mM, pH 7.4 |
| Mes-TEA | MES 100mM, TEA 100mM, pH 6 |

**Table S4. List of antibodies used in this work.**

| Name | reference | Host specie | Classification |
| --- | --- | --- | --- |
| Tau (human) monoclonal antibody (tau12) | Enzo Life Science ENZ-ABS216-0100 | mouse | primary |
| phospho-tau (ser199,ser202) | Invitrogen 44-768G | rabbit | primary |
| phospho-tau (ser214) | Invitrogen 44-742G | rabbit | primary |
| GFP antibody (b-2) | Santa Cruz Biotechnology | mouse | primary |
| pgk1 monoclonal antibody (22c5d8) | Invitrogen 459250 | mouse | primary |
| mdm38 | Walter Naupert's Lab | rabbit | primary |
| tom20 | Walter Naupert's Lab | rabbit | primary |
| goat Anti-rabbit igg (H+l) Cross-Adsorbed Secondary Antibody hrp | Invitrogen CAT G-21234 | goat | secondary |
| Peroxidase-Conjugated AffiniPure Goat Anti-Mouse IgG (h+l) | Jackson InmunoResearch  CAT 115-035-003 | goat | secondary |
