# Supplementary material for: Tau Protein Disrupts Mitochondrial Homeostasis in a Yeast Model: Implications for Alzheimer’s Disease
Source: Mol Neurobiol. 2025 Aug 8;62(12):16460–71. doi: 10.1007/s12035-025-05255-z (PMC12559152; doi:10.1007/s12035-025-05255-z)
Supplement: Supplementary file 3 — Supplementary file3 (DOCX 23.1 MB) [file 12035_2025_5255_MOESM3_ESM.docx]

**Tau protein disrupts mitochondrial homeostasis in a yeast model: implications for Alzheimer’s disease**

Yaisa Castillo-Casaña^1^, Laura Kawasaki^1^, Clorinda Arias^2^, Hilario Ruelas-Ramírez^3^, Soledad Funes^3^, Norma Silvia Sánchez^3^, María Guadalupe Códiz-Huerta^4^, Laura Ongay-Larios^4^ and Roberto Coria^1^*.

Supplementary original figures and blots:

Original figure 1A


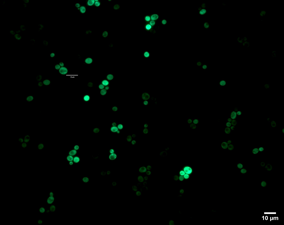

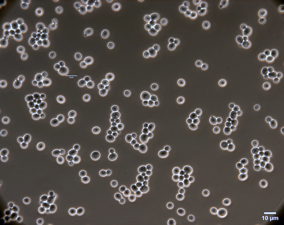

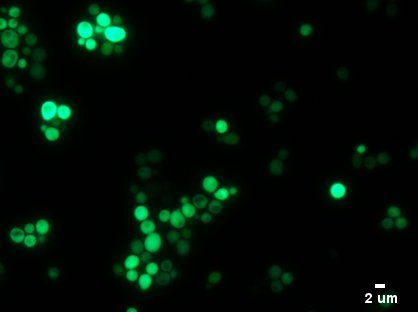

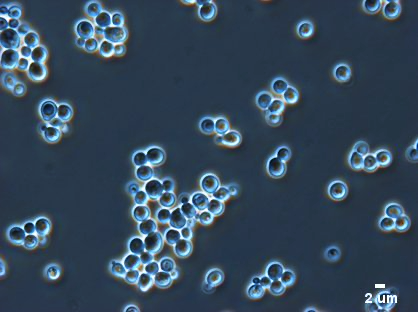


Original blots Figure 1B

KDa

MW

MW

KDa

MW

1

2

3

6

days

1

2

3

6

days

-Tau

-Tau

2

3

6

days

1


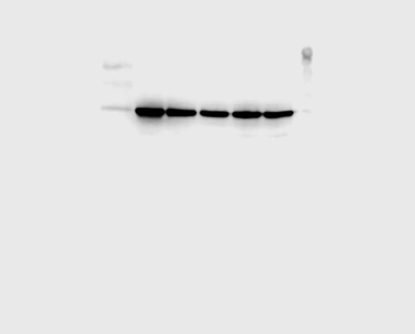

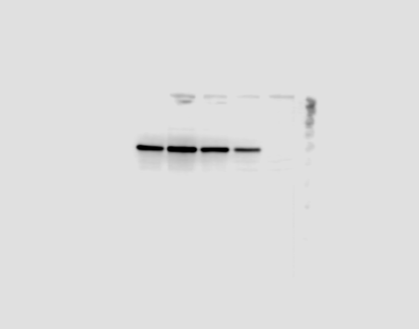

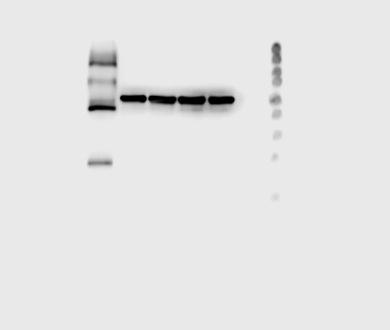


KDa

-Tau

180

130

130

180

100

70

55

130

100

180

55

70

Pgk1

Tau

25

40

P-Tau

70

55

40

100

35

15

40

15

25

25

15

Original Figure 2A


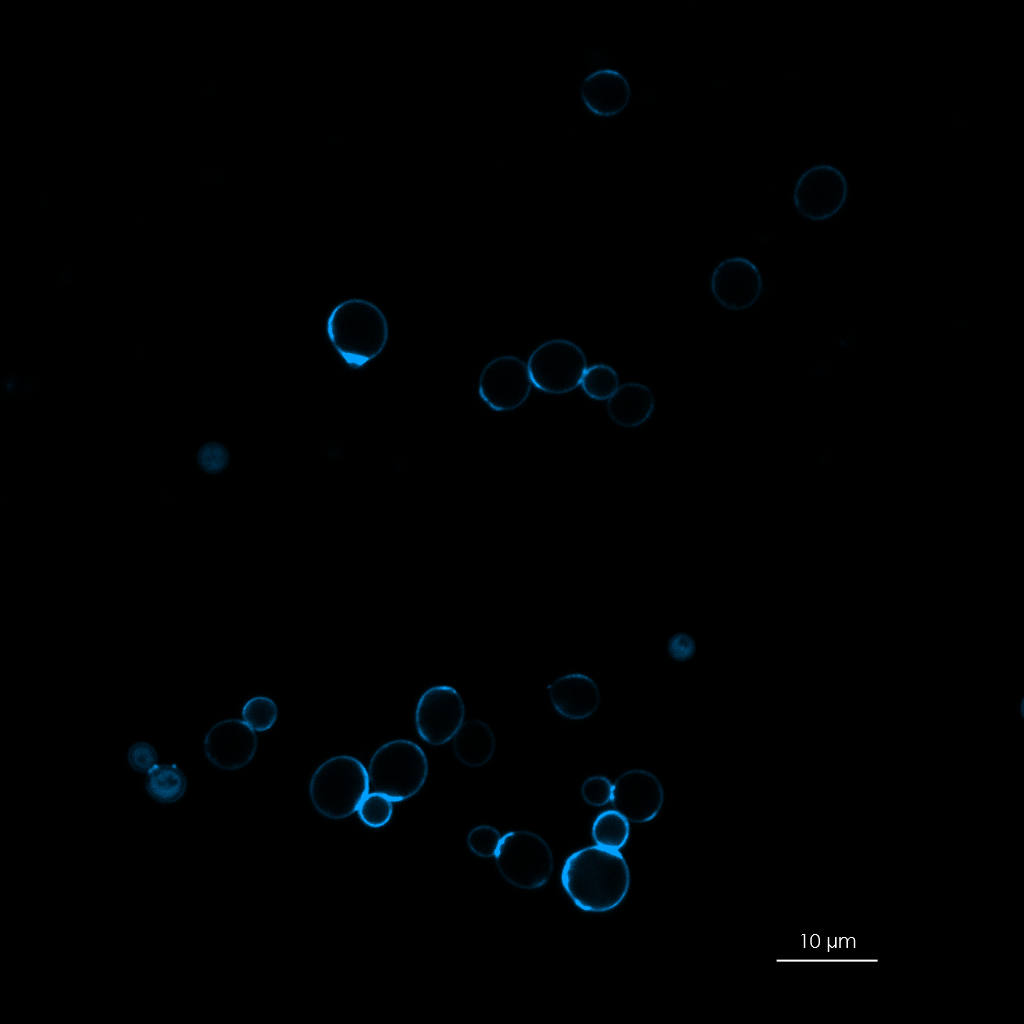

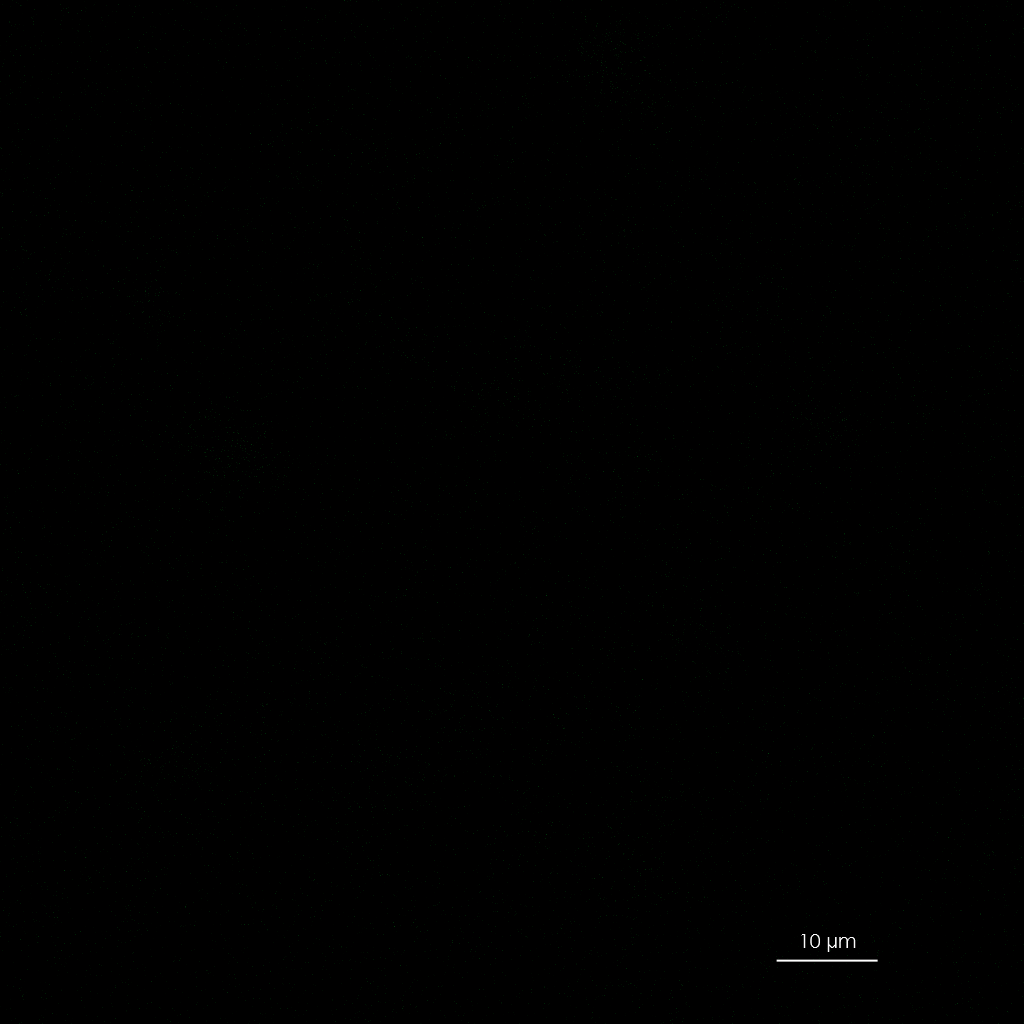

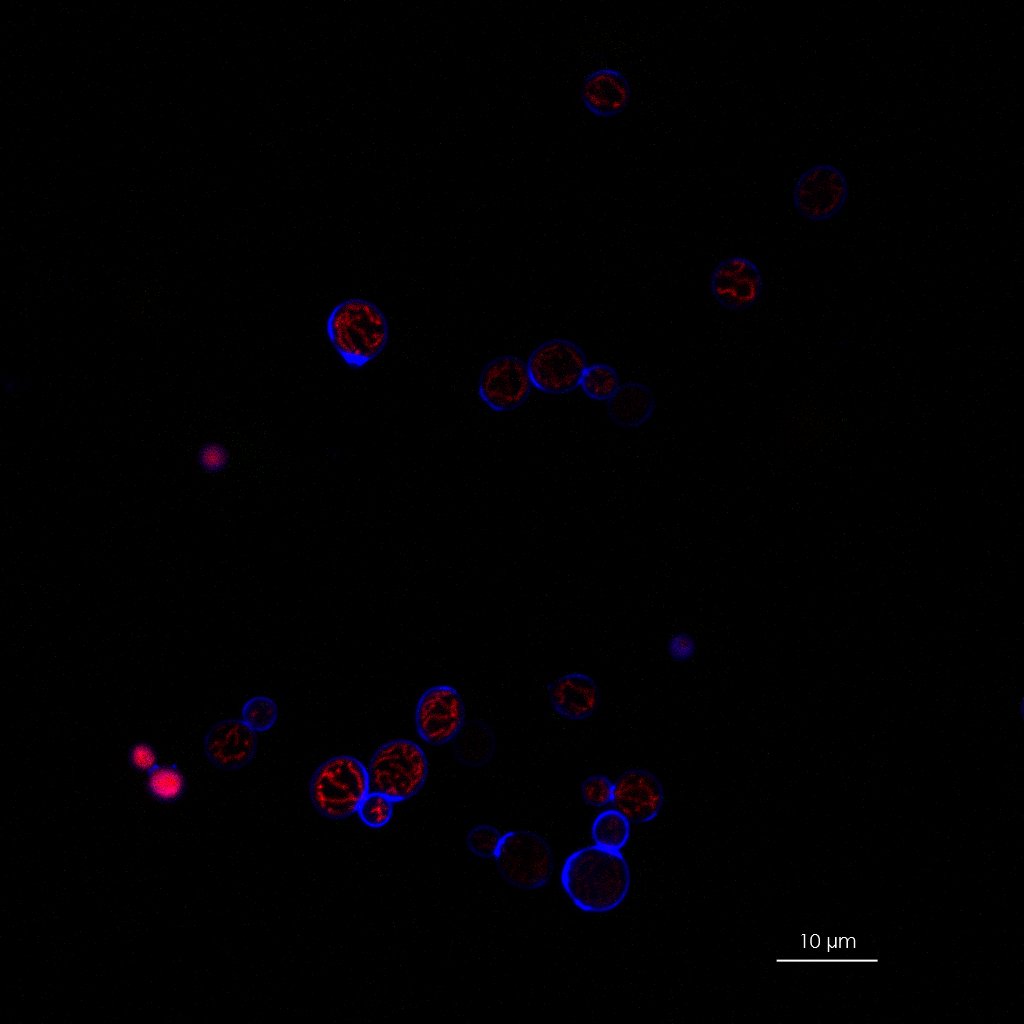

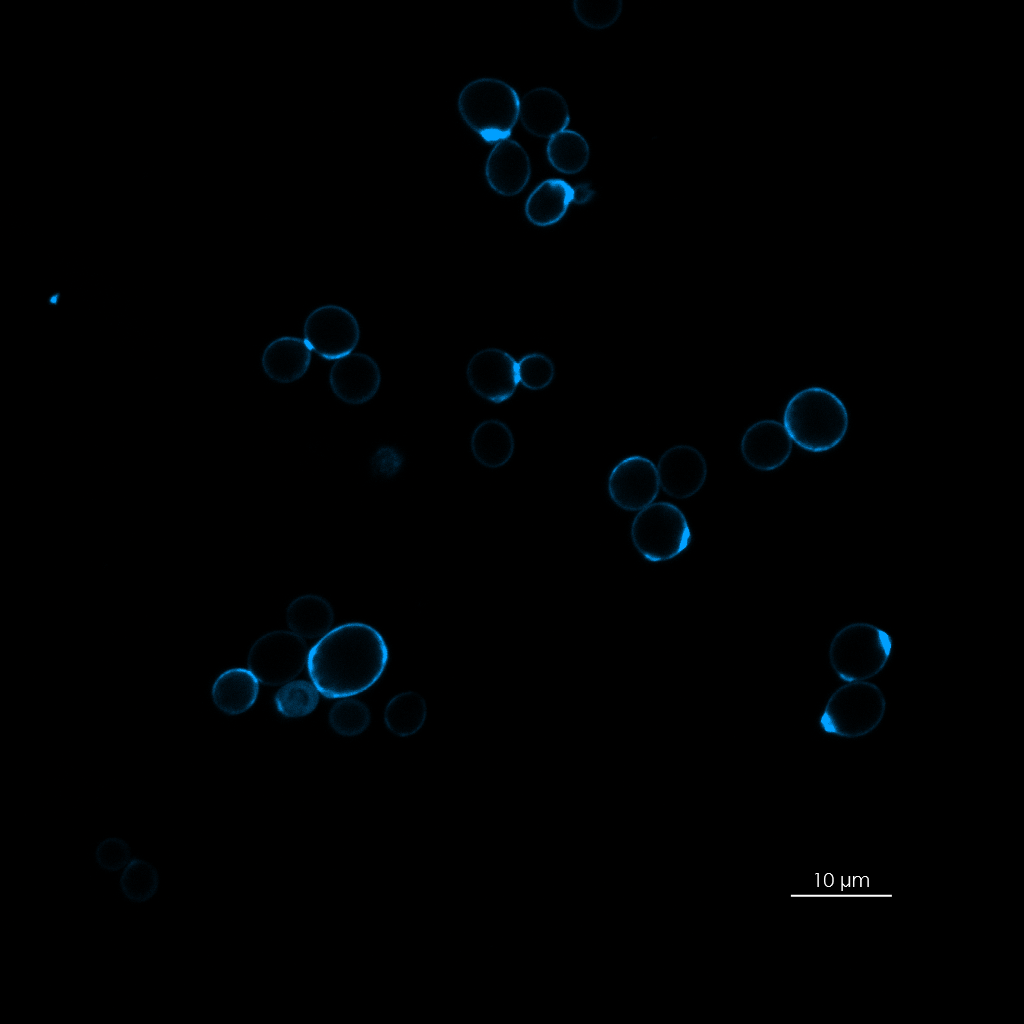

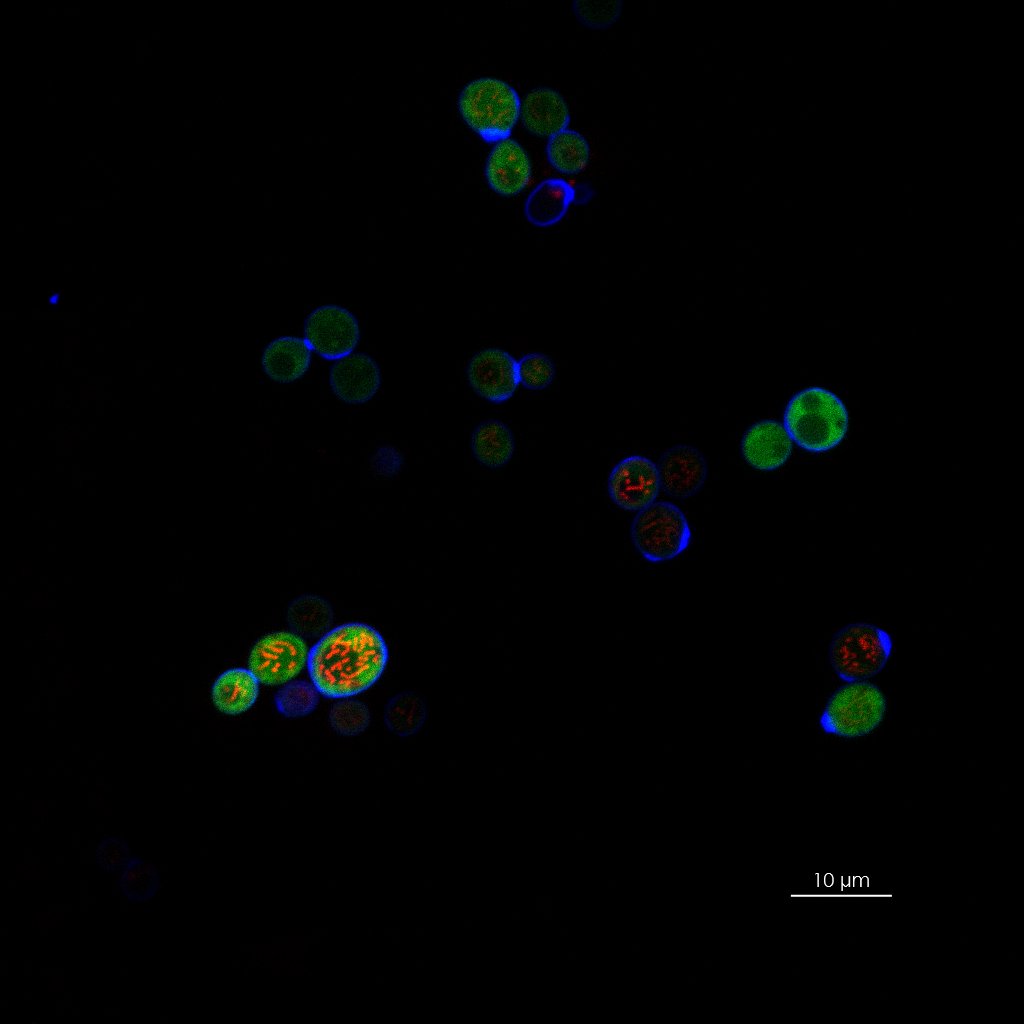

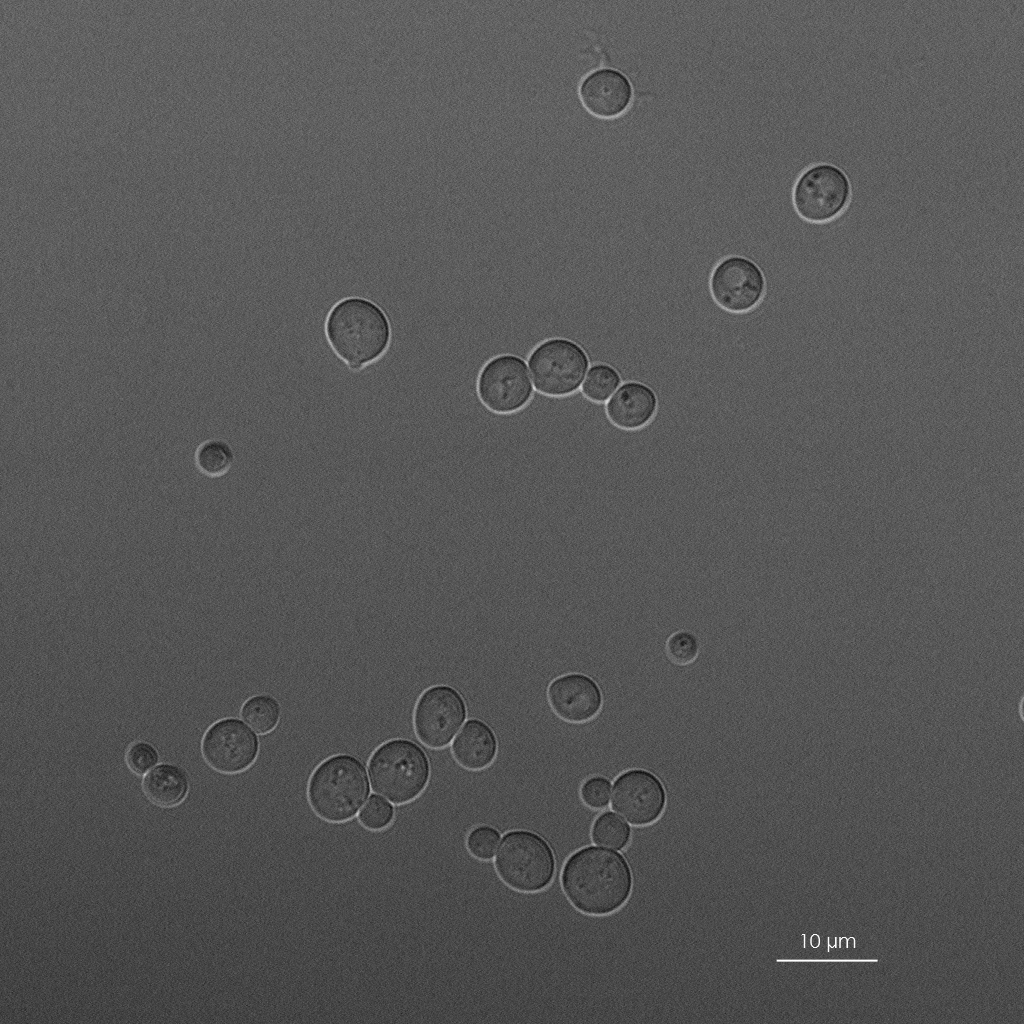

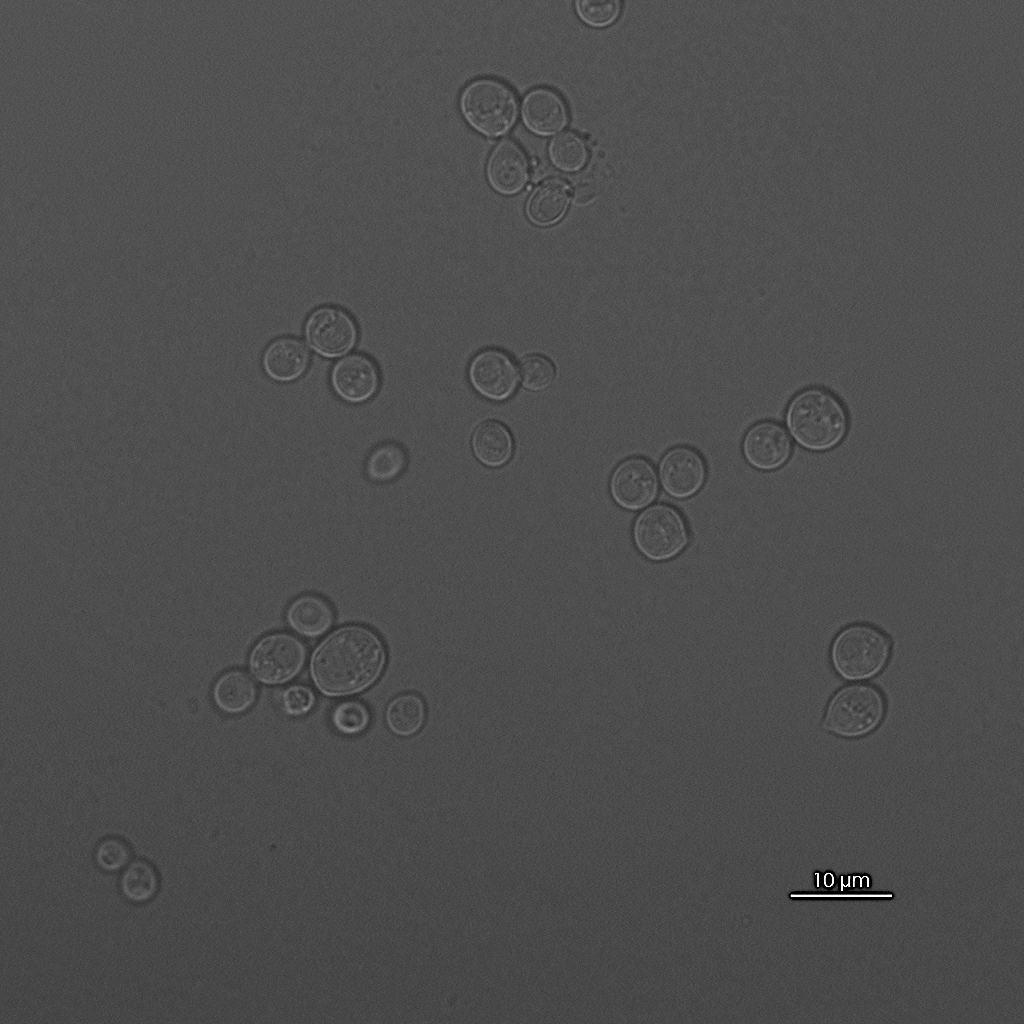

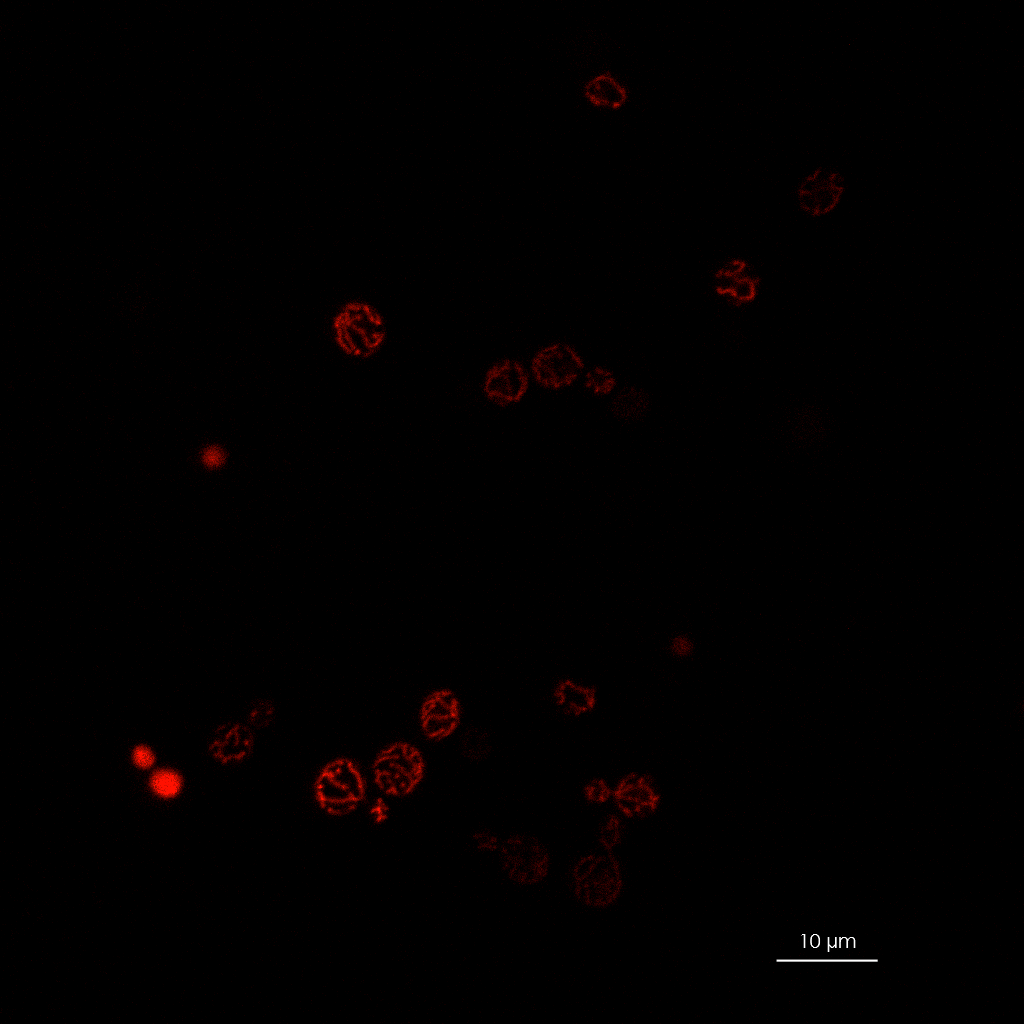

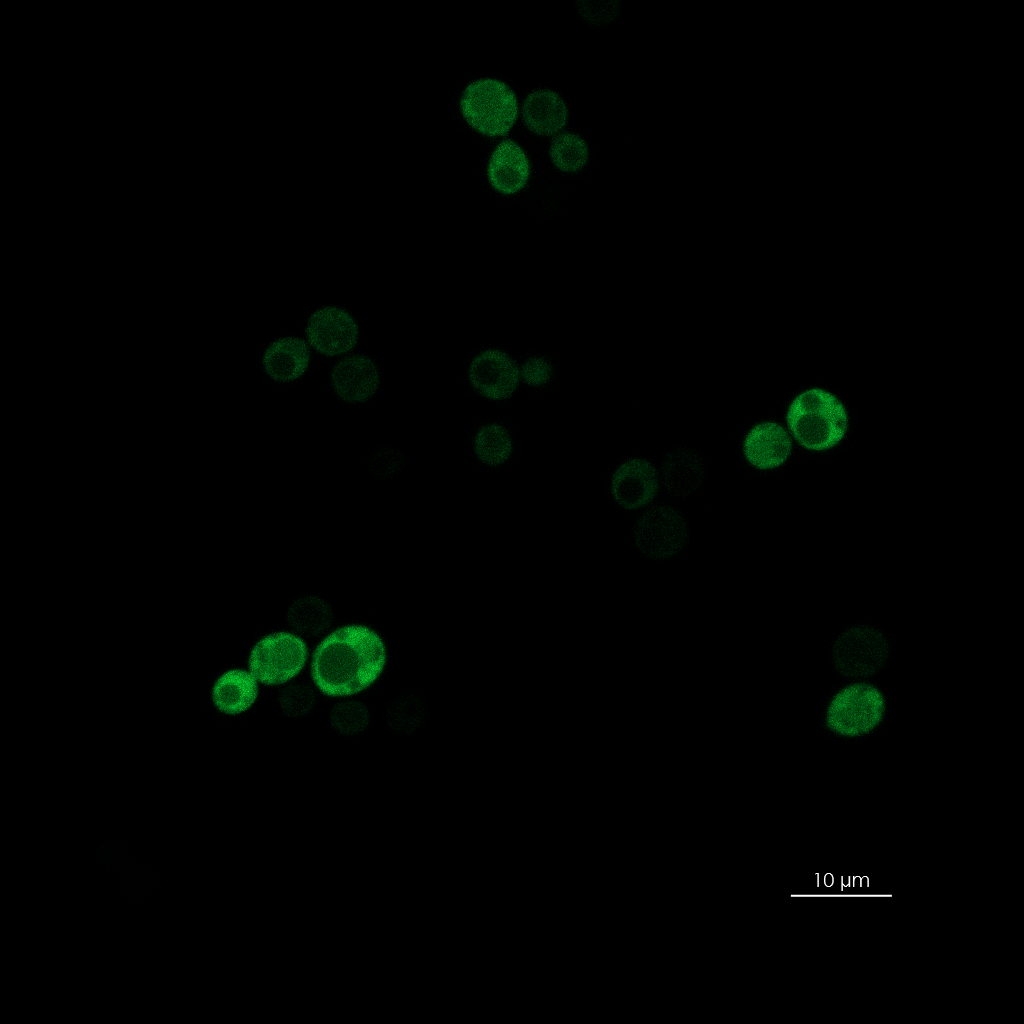

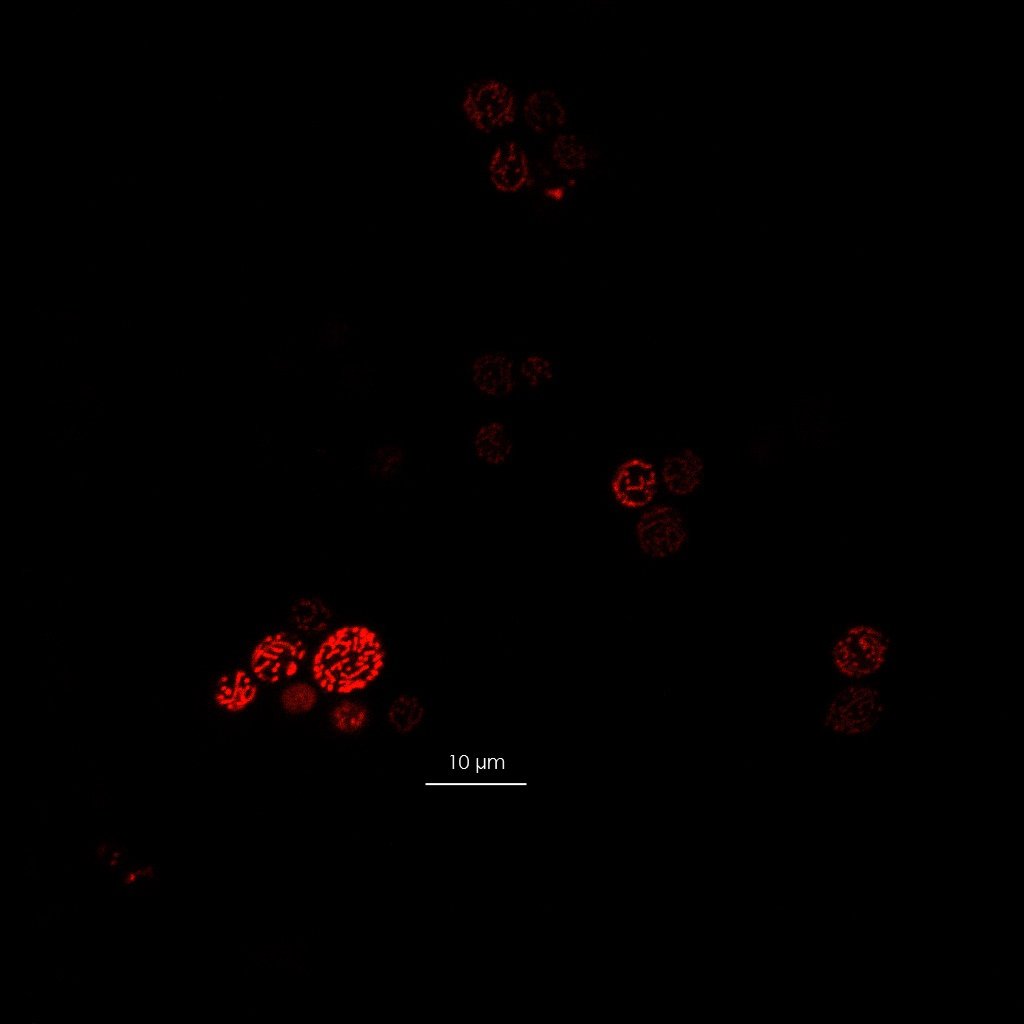


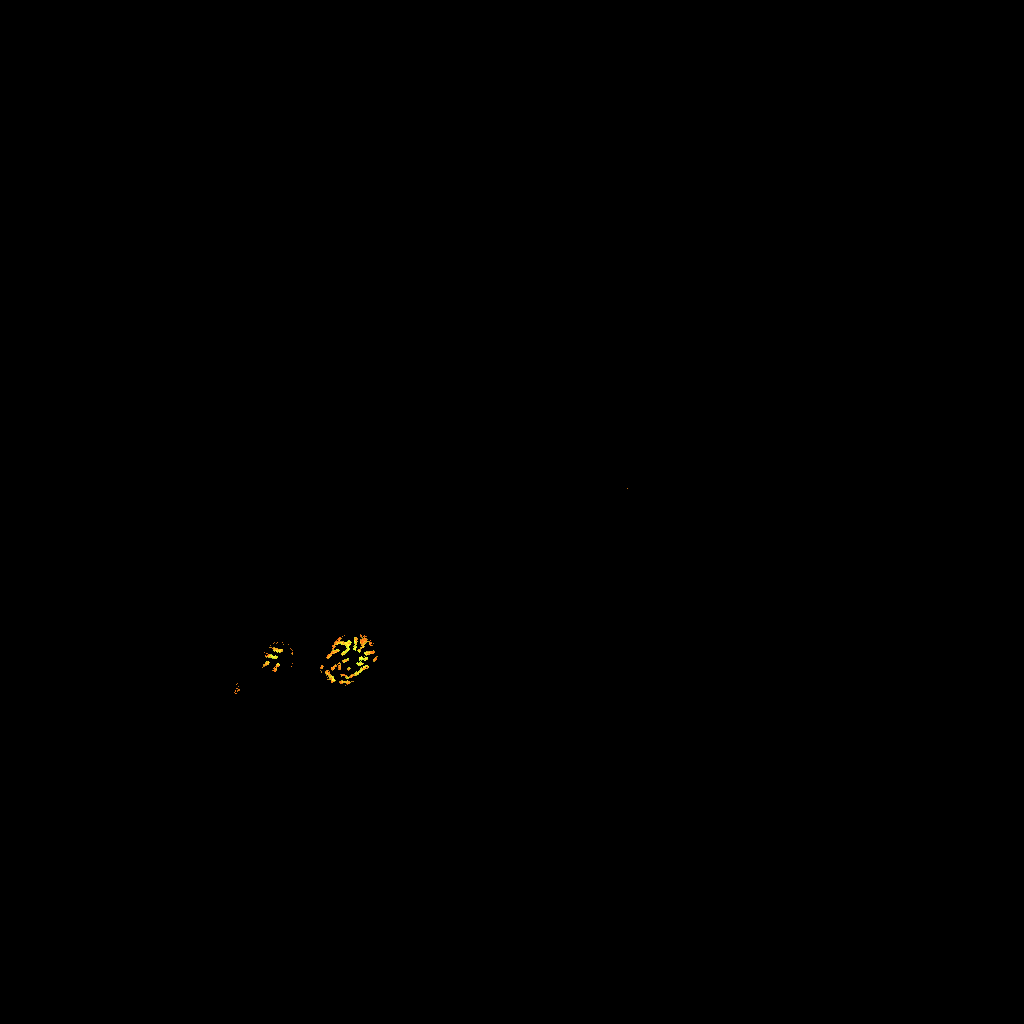

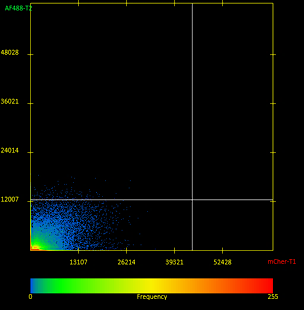


Original blots Figure 2B


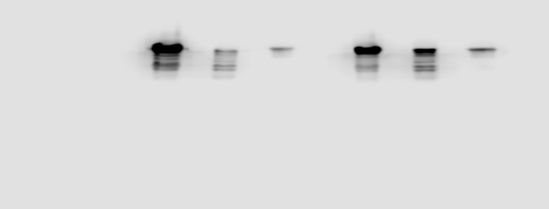

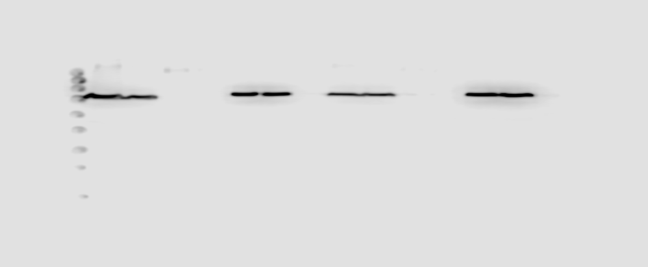

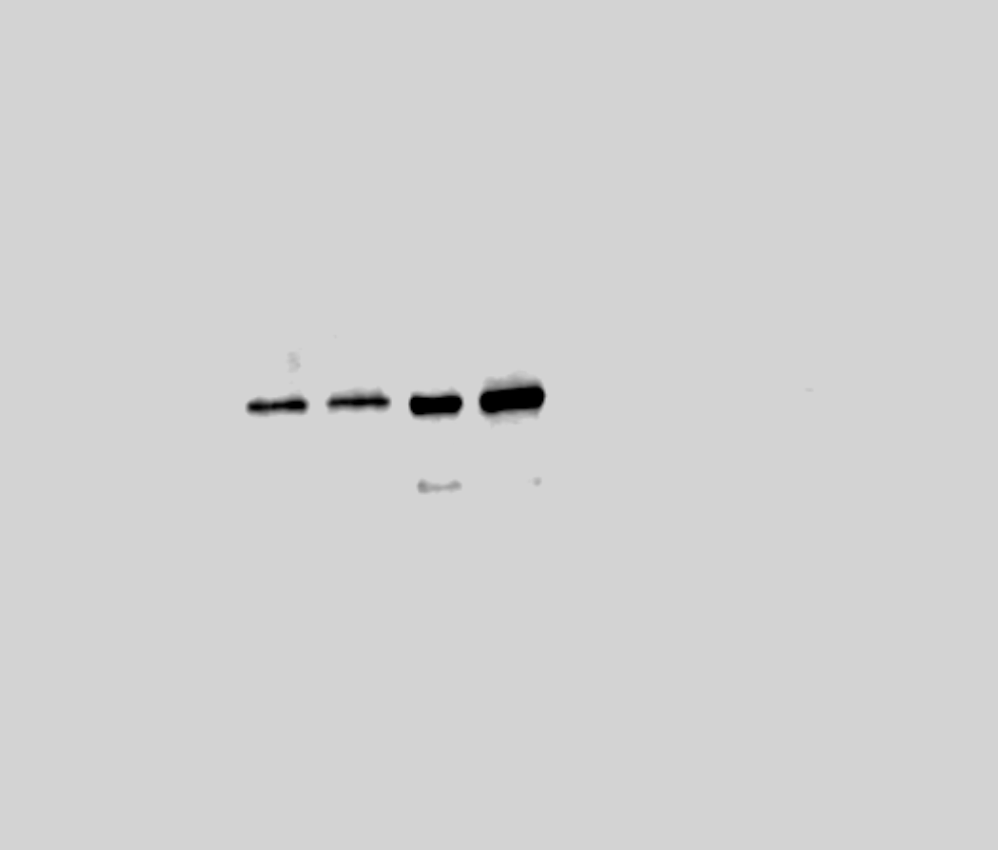


KDa

-

-

-

-

-

-

+

+

+

+

+

+

T

Cy

Mt

T

Cy

Mt

+

+

+

T

Cy

Mt

-

-

-

+

+

+

T

Cy

Mt

-

-

-

+

+

+

T

Cy

Mt

-

-

-

Tau

KDa

180

55

70

100

130

180

Tau

130

100

70

55

35

40

Mdm38

40

35

15

25

15

25

KDa

180

40

55

70

100

130

Pgk1

25

35

15

Original blots Figure 2C


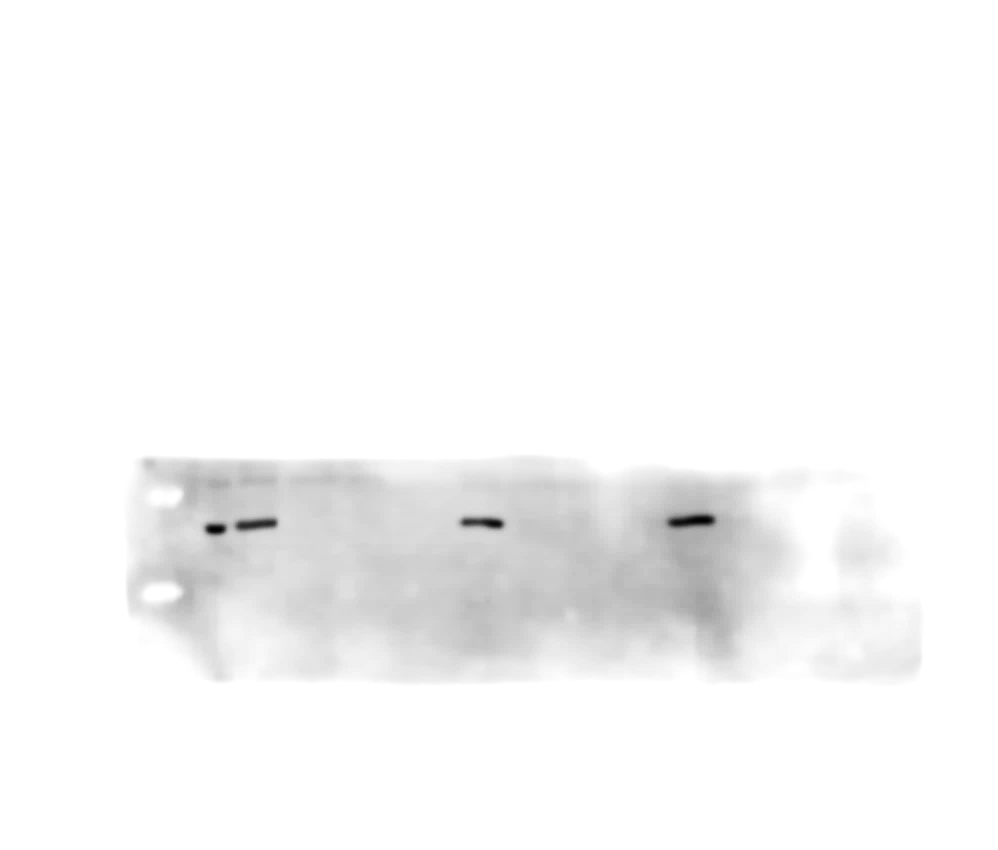

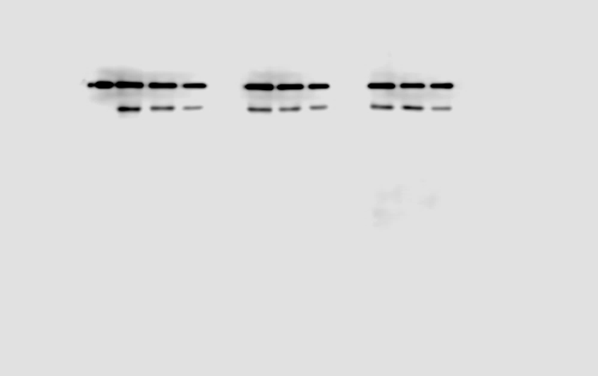

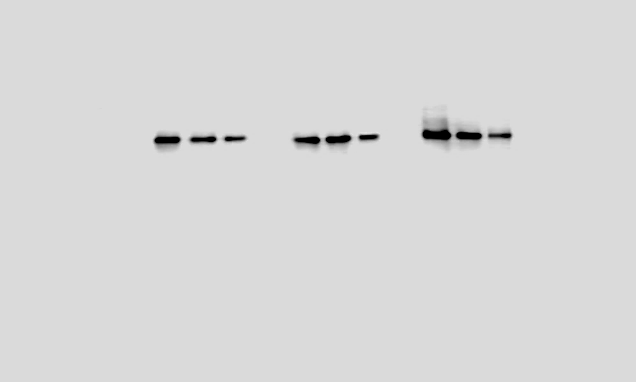


replica

replica

Mp

Mt

+

+

+

+

-

replica

replica

Mp

Mt

+

+

+

+

-

replica

replica

Mp

Mt

+

+

+

+

-

KDa

Tau

180

130

100

70

55

40

Tau

35

25

15

KDa

100

130

180

55

70

Mdm38

25

35

40

15

KDa

25

Tom20

15

Original blots Figure 3


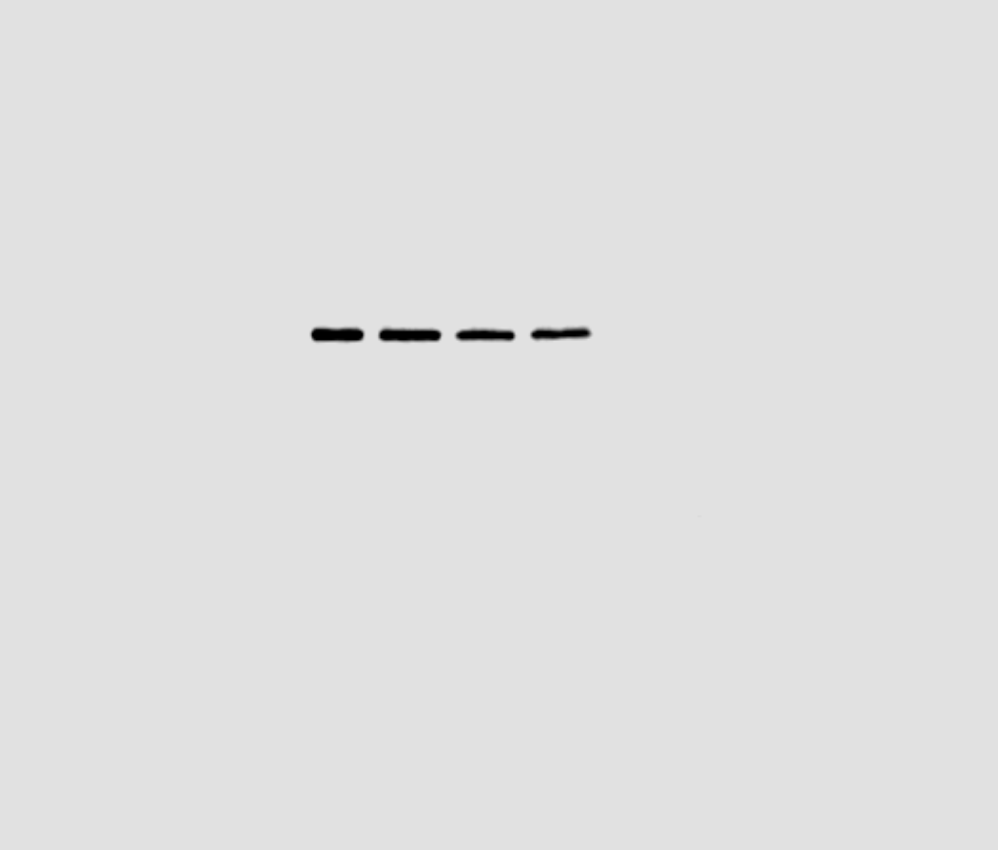


Δhsp104

replica

wt

Δydj1

Δssa1


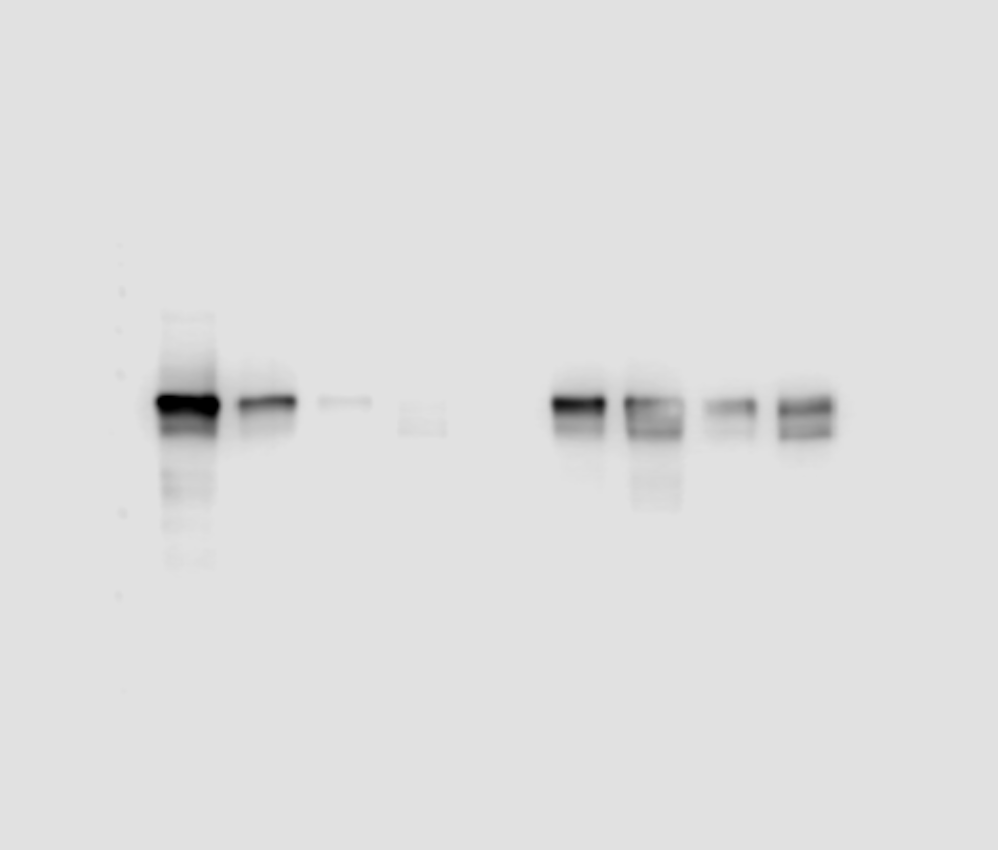


KDa

70

100

130

180

Tau

35

40

55

15

25

KDa

70

100

130

180

Mdm38

35

40

55

15

25


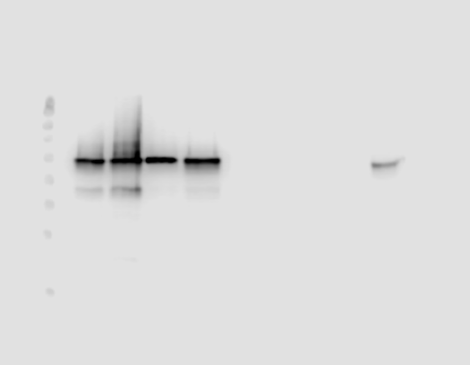

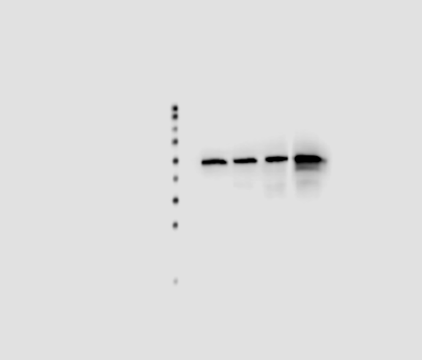


Δydj1

Δssa1

wt

Δhsp104

Δssa1

Δydj1

180

130

100

Tau

Cytoplasm

KDa

KDa

70

55

40

35

25

15

15

25

35

40

55

70

100

130

180

Total fraction

Δhsp104

wt

Original Figure 4A


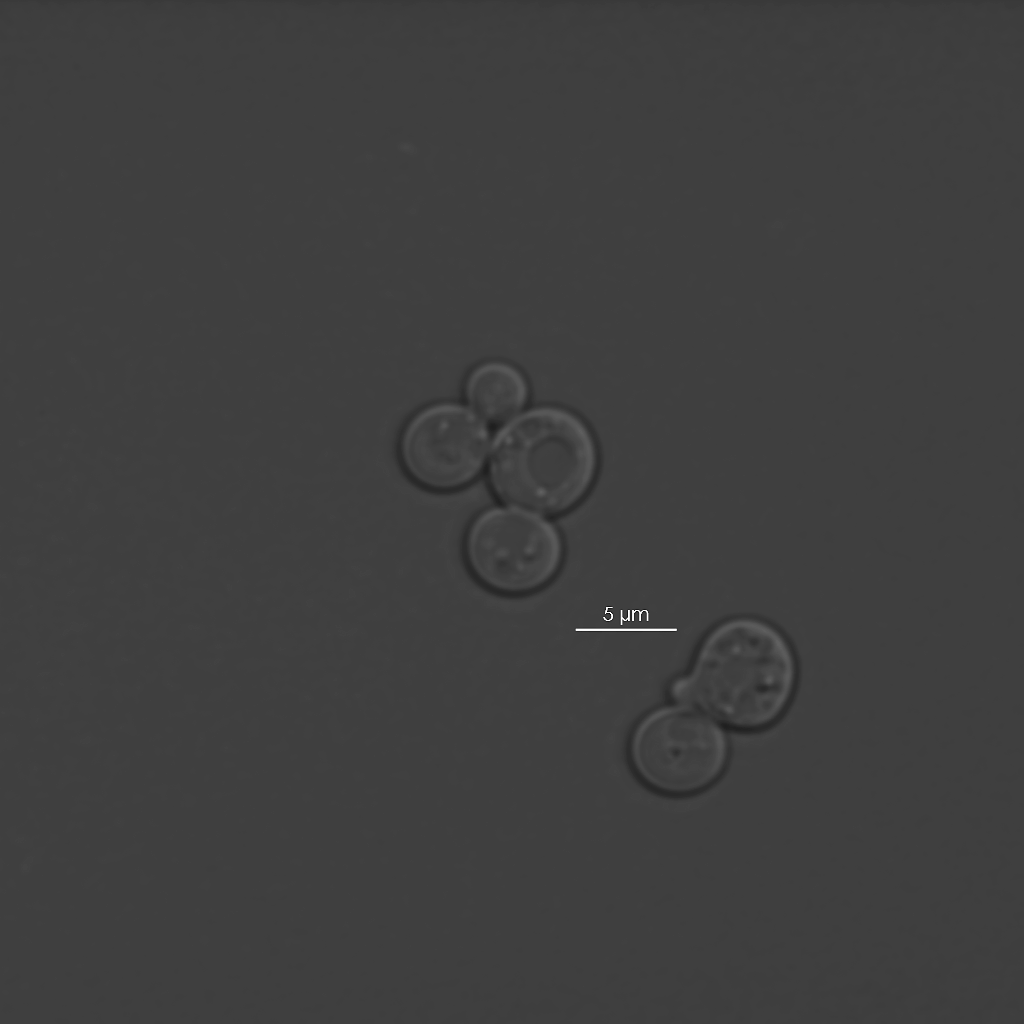

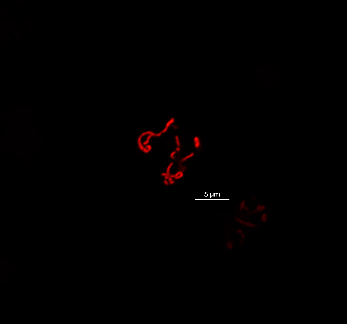

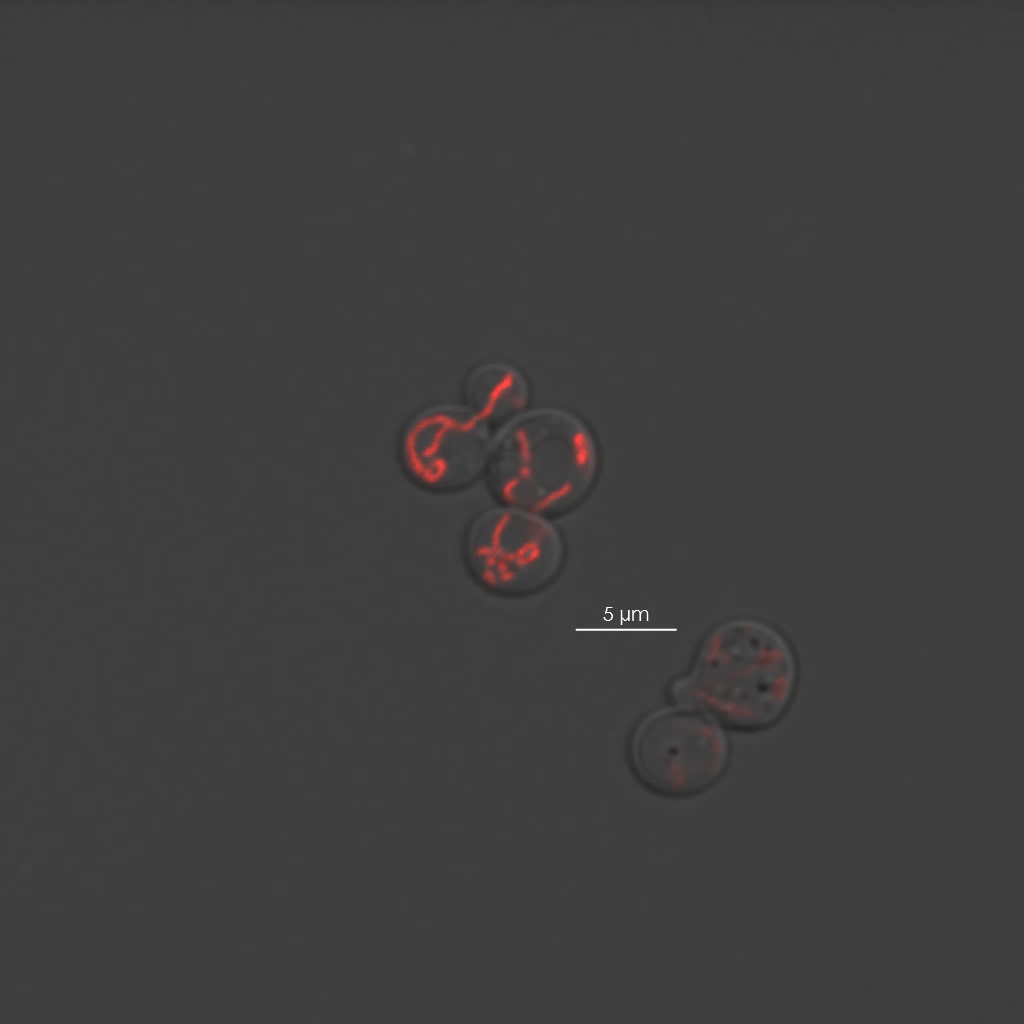


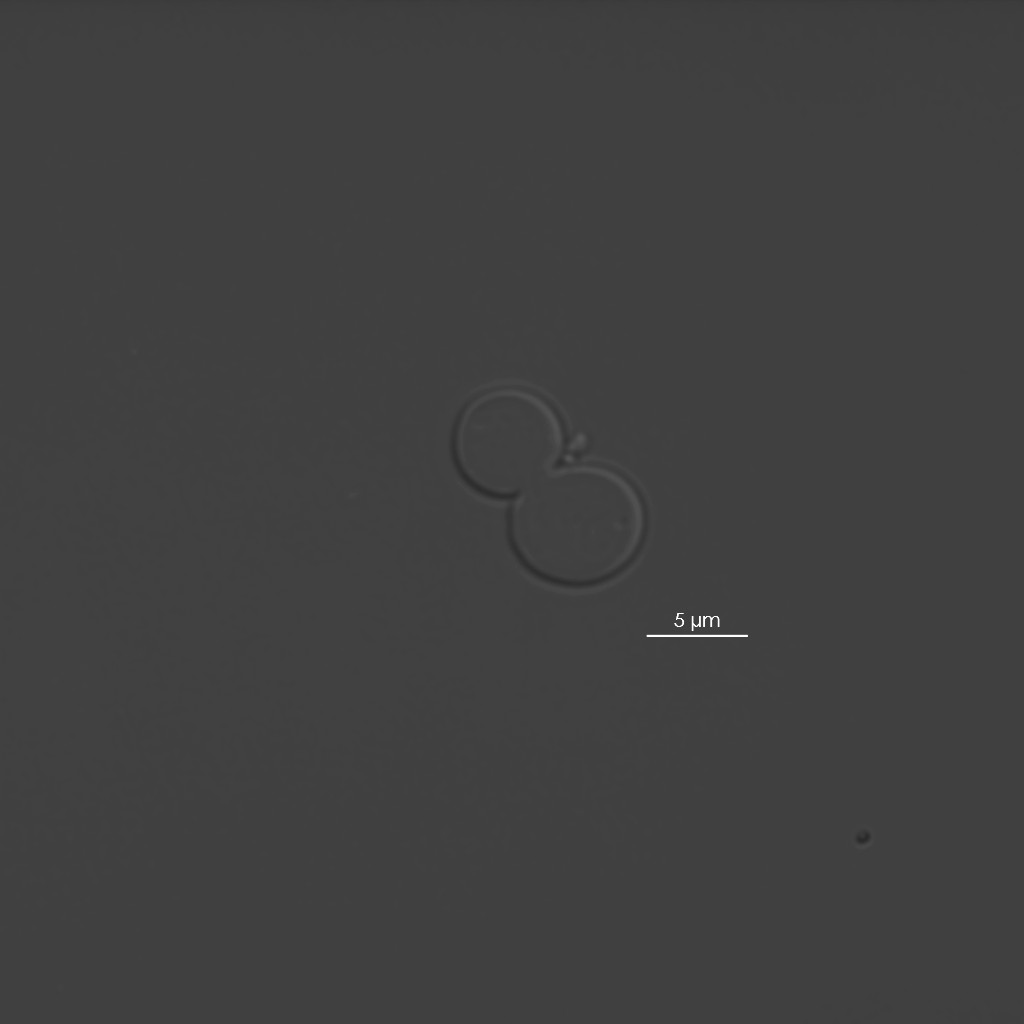

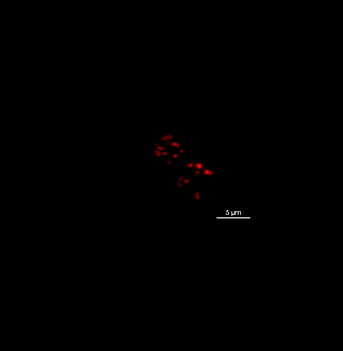

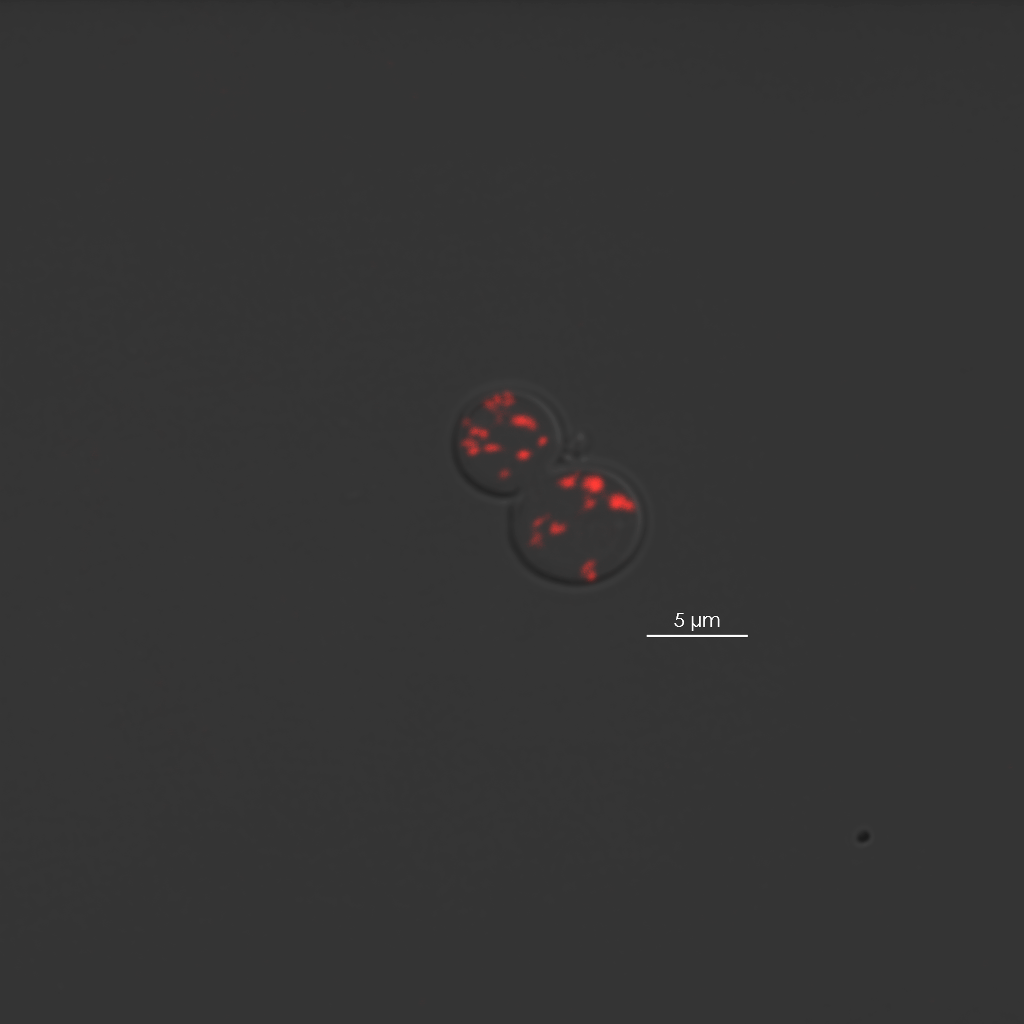


Original Figure 5


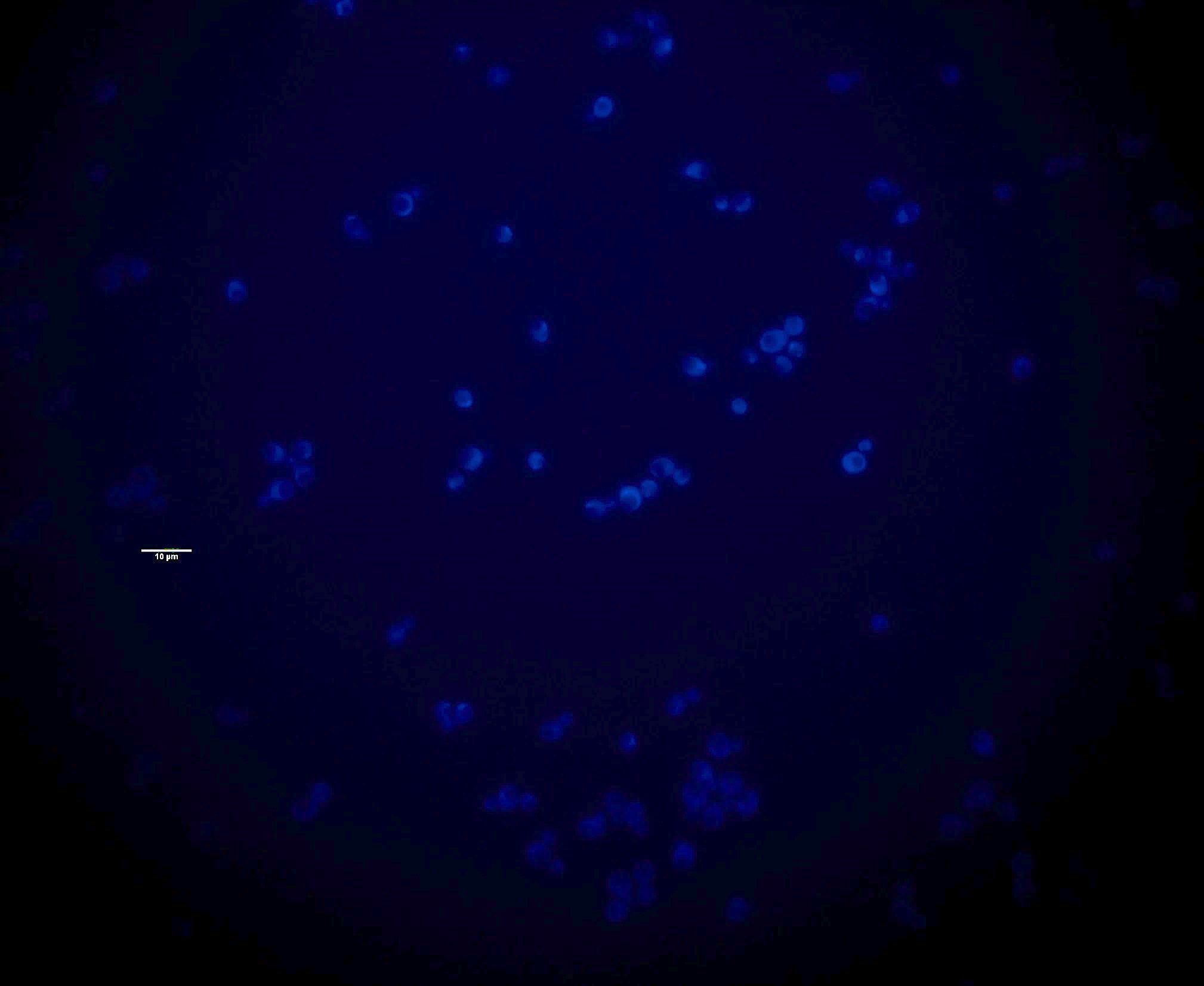

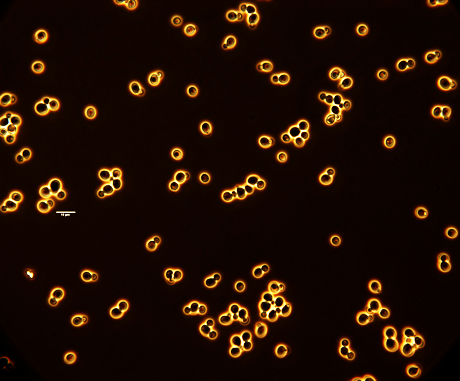

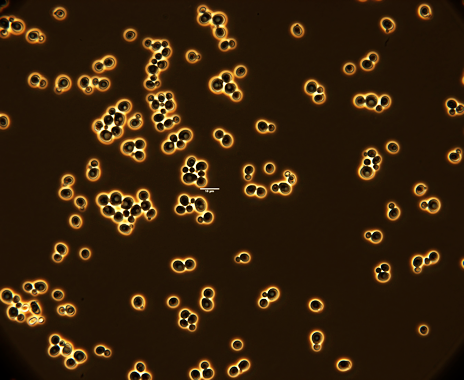


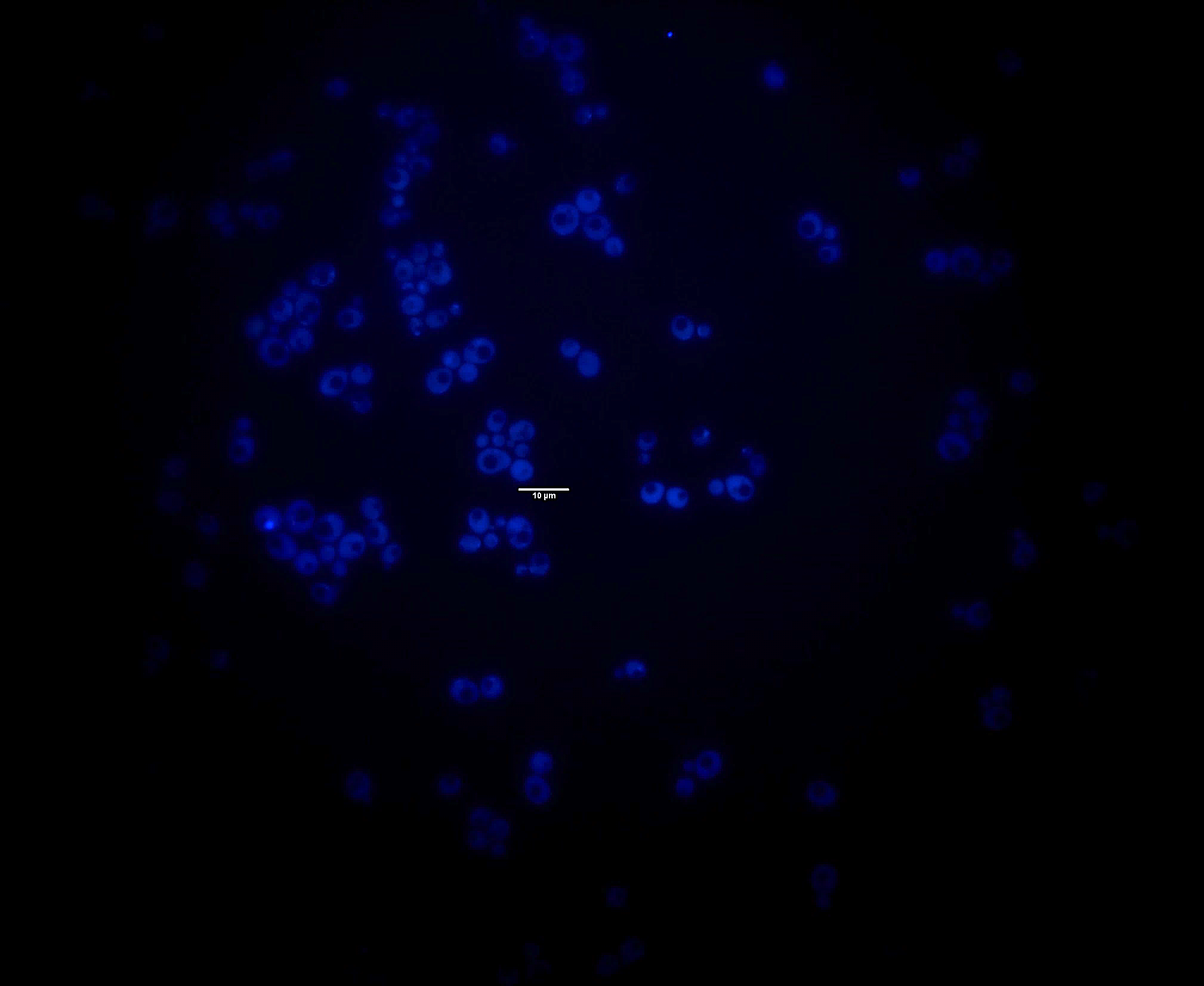


Original blots Figure 6A


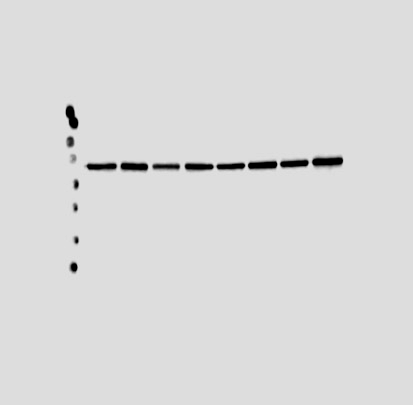

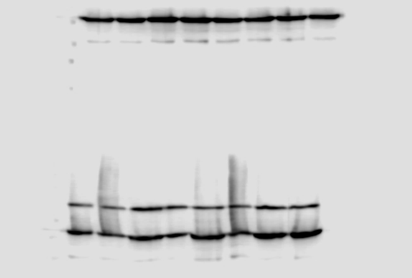

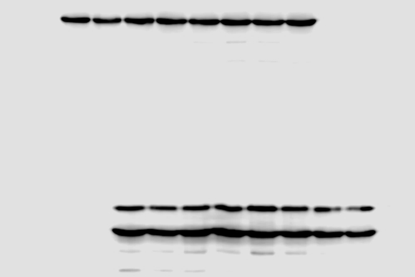

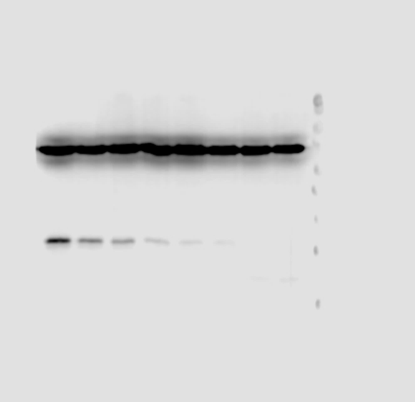


atg32

4

3

2

0

+

-

+

-

+

-

+

-

wt

4

3

2

0

+

-

+

-

+

-

+

-

Time h

Time h

KDa

KDa

Tau

Tau

130

180

180

130

100

70

100

70

55

Idh1-GFP

40

55

40

35

35

25

GFP

25

15

KDa

Pgk1

40

40

Pgk1

Original blots Figure 6B


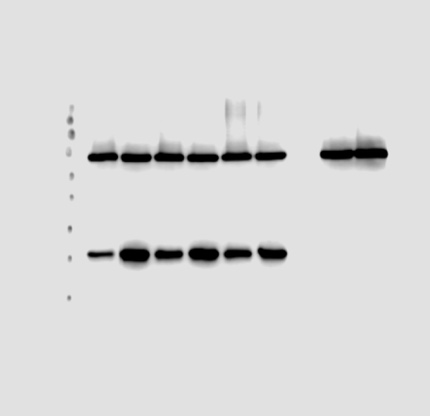

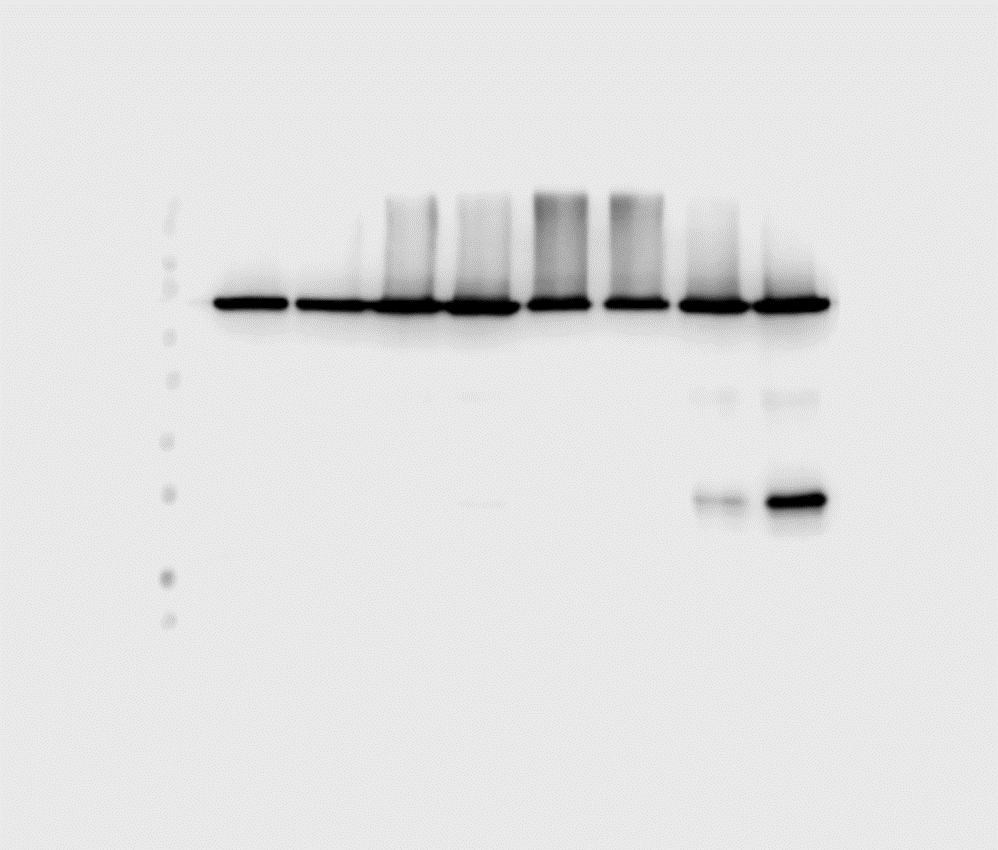


wt

atg32

3

6

5

4

+

-

+

-

+

-

+

-

wt

3

2

1

0

+

-

+

-

+

-

+

-

Tau

Time days

Tau

Time days

KDa

KDa

130

180

130

180

70

100

70

100

Idh1-GFP

40

55

40

55

35

25

35

GFP

15

25

15


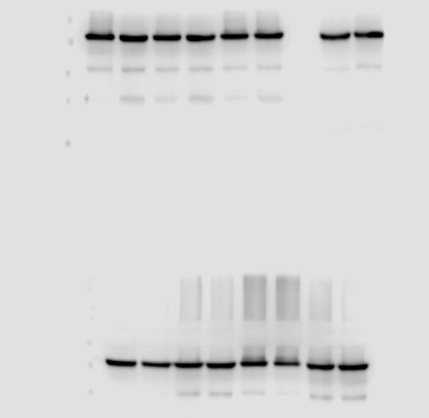


Pgk1

40

40

Pgk1

Original blots Figure 7

replica


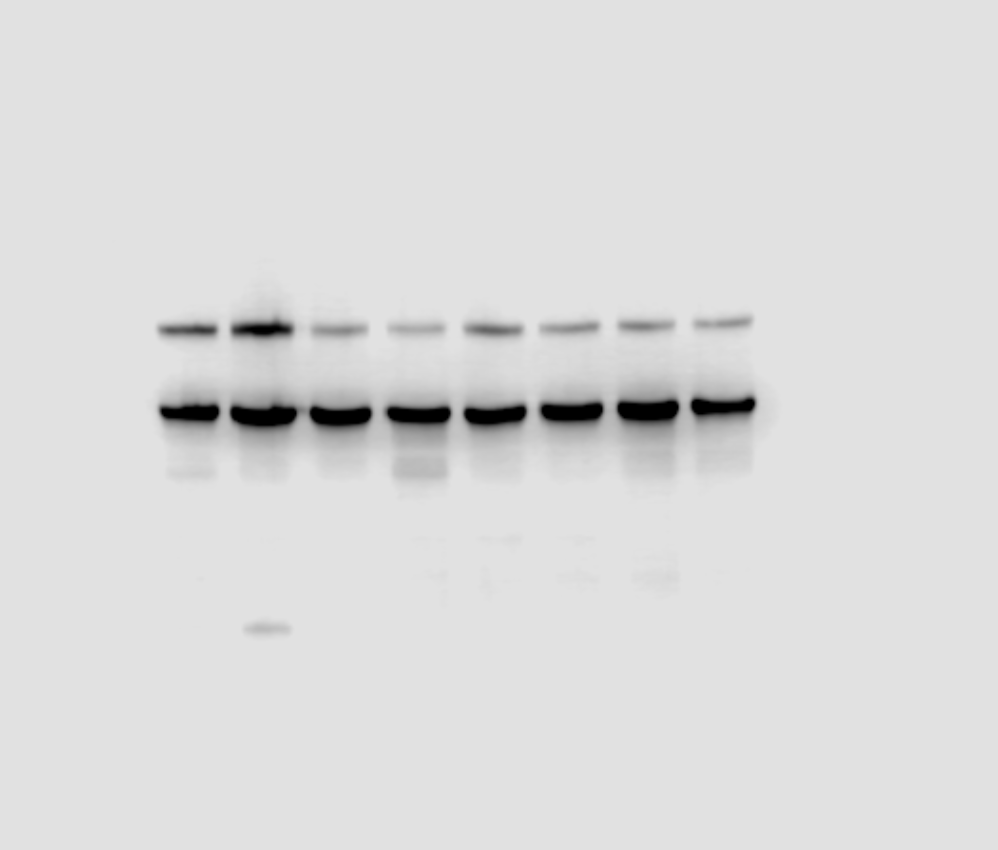

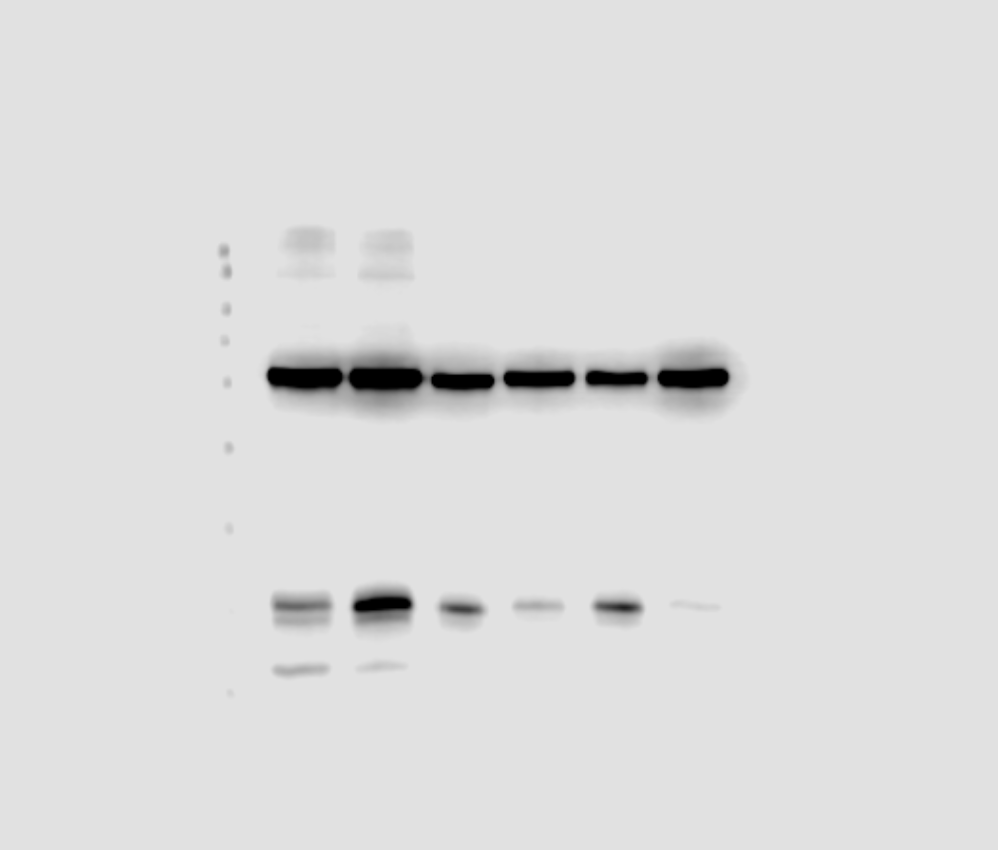

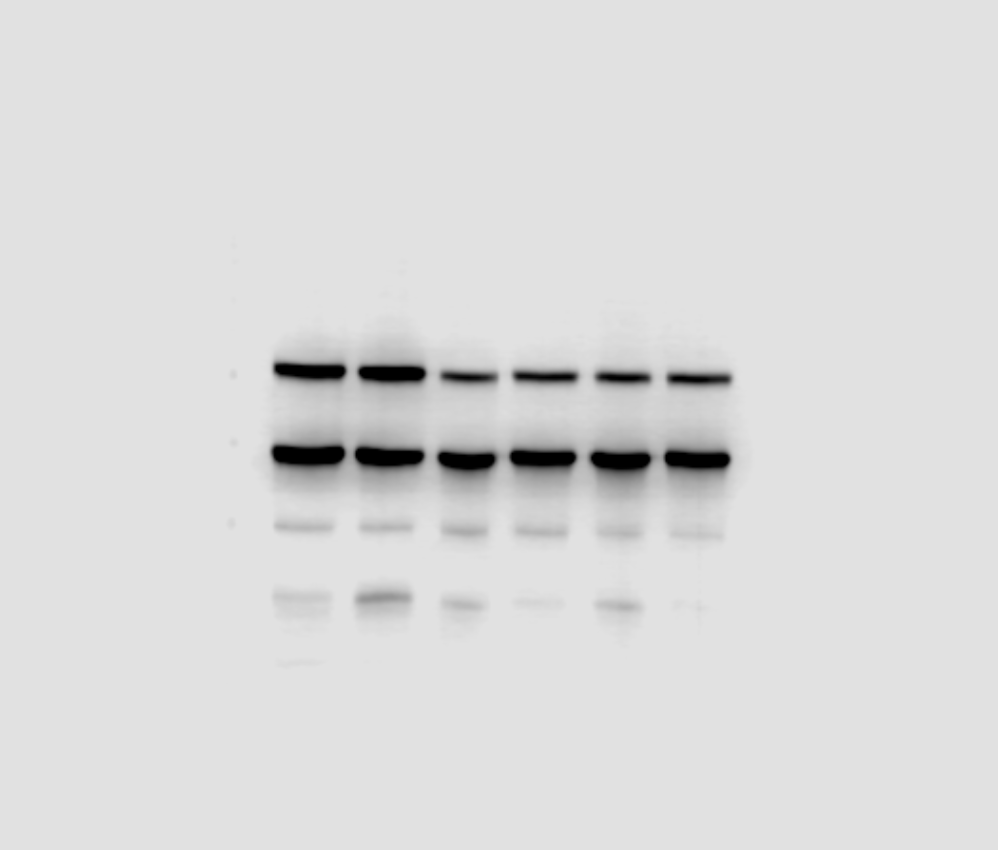

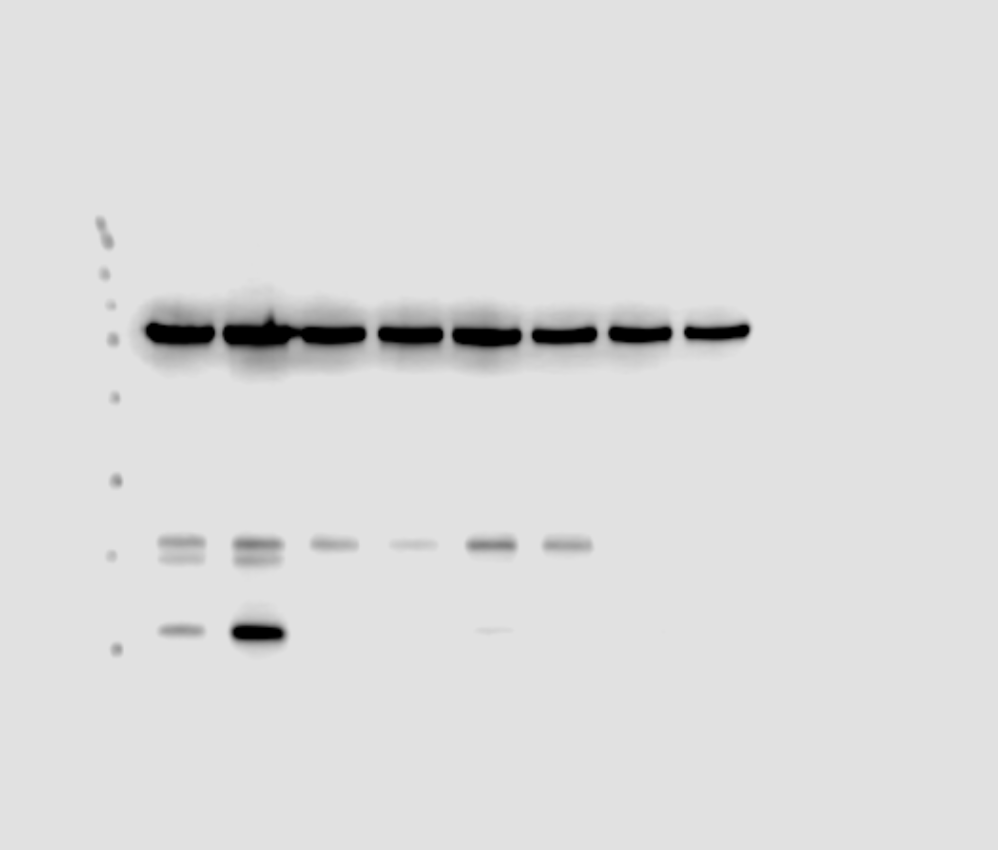


40

40

Pgk1

180

130

100

70

55

40

35

25

15

25

35

40

55

70

100

130

180

KDa

KDa

GFP

Idh1-GFP

Tau

atg32

rtg3

rtg1

wt

rtg3

rtg1

wt

+

-

+

-

+

-

+

-

+

-

+

-

+

-
